# Exploiting biological priors and sequence variants enhances QTL discovery and genomic prediction of complex traits

**DOI:** 10.1186/s12864-016-2443-6

**Published:** 2016-02-27

**Authors:** I. M. MacLeod, P. J. Bowman, C. J. Vander Jagt, M. Haile-Mariam, K. E. Kemper, A. J. Chamberlain, C. Schrooten, B. J. Hayes, M. E. Goddard

**Affiliations:** Faculty of Veterinary & Agricultural Science, University of Melbourne, Victoria, 3010 Australia; Dairy Futures Cooperative Research Centre, AgriBio, Bundoora, Victoria Australia; AgriBio, Dept. Economic Development, Jobs, Transport & Resources, Victoria, Australia; Biosciences Research Centre, La Trobe University, Victoria, Australia; CRV, 6800 AL Arnhem, The Netherlands

**Keywords:** Bayesian analysis, Biological model, Genomic selection, Whole-genome association analysis, Milk traits, Dairy cattle

## Abstract

**Background:**

Dense SNP genotypes are often combined with complex trait phenotypes to map causal variants, study genetic architecture and provide genomic predictions for individuals with genotypes but no phenotype. A single method of analysis that jointly fits all genotypes in a Bayesian mixture model (BayesR) has been shown to competitively address all 3 purposes simultaneously. However, BayesR and other similar methods ignore prior biological knowledge and assume all genotypes are equally likely to affect the trait. While this assumption is reasonable for SNP array genotypes, it is less sensible if genotypes are whole-genome sequence variants which should include causal variants.

**Results:**

We introduce a new method (BayesRC) based on BayesR that incorporates prior biological information in the analysis by defining classes of variants likely to be enriched for causal mutations. The information can be derived from a range of sources, including variant annotation, candidate gene lists and known causal variants. This information is then incorporated objectively in the analysis based on evidence of enrichment in the data. We demonstrate the increased power of BayesRC compared to BayesR using real dairy cattle genotypes with simulated phenotypes. The genotypes were imputed whole-genome sequence variants in coding regions combined with dense SNP markers. BayesRC increased the power to detect causal variants and increased the accuracy of genomic prediction. The relative improvement for genomic prediction was most apparent in validation populations that were not closely related to the reference population. We also applied BayesRC to real milk production phenotypes in dairy cattle using independent biological priors from gene expression analyses. Although current biological knowledge of which genes and variants affect milk production is still very incomplete, our results suggest that the new BayesRC method was equal to or more powerful than BayesR for detecting candidate causal variants and for genomic prediction of milk traits.

**Conclusions:**

BayesRC provides a novel and flexible approach to simultaneously improving the accuracy of QTL discovery and genomic prediction by taking advantage of prior biological knowledge. Approaches such as BayesRC will become increasing useful as biological knowledge accumulates regarding functional regions of the genome for a range of traits and species.

**Electronic supplementary material:**

The online version of this article (doi:10.1186/s12864-016-2443-6) contains supplementary material, which is available to authorized users.

## Background

In humans, plants and livestock, data on genome-wide SNP markers and complex trait phenotypes have been used for 3 purposes: to identify SNP associated with the trait, to study the genetic architecture of the trait, and to predict the genetic value or future phenotype of individuals. Although different statistical methods are commonly used for these three purposes, the Bayesian “genomic selection” or “genomic prediction” approach of Meuwissen et al [1] can be effectively used for all 3 purposes in a single analysis [2, 3]. This Bayesian approach fits the effects of all SNP simultaneously in the statistical model assuming that they are random effects drawn from a distribution. Erbe et al [4] modified the approach of [1], proposing a mixture of normal distributions to model the SNP effects. Their model allows many effects to be zero but some effects to be relatively large and is flexible enough to cover a range of distributions that might apply to different traits. They called the method BayesR. In both human and livestock data, BayesR has been demonstrated to be equal or superior to linear mixed model methods, such as GBLUP (genomic best linear unbiased prediction), for genomic prediction and QTL mapping [2, 3, 5].

To date, methods such as BayesR, GBLUP and traditional GWAS (genome wide association studies) assume that each variant is equally likely to affect the trait: that is, no prior biological knowledge is included in the model. Instead, the available biological knowledge is often applied post-analysis, in a somewhat arbitrary and potentially biased manner to confirm candidate genes and mutations. When analysing dense SNP array genotypes it is reasonable to assume a model in which each marker may equally affect the trait. However, this assumption is less sensible when analysing whole-genome sequence variants, some of which may be known to cause non-synonymous coding changes or affect regulatory regions of candidate genes. In humans, as well as some livestock it is now possible to impute sequence variants for many thousands of individuals, so there is a need to develop methods that objectively include independent biological information in the analytical model.

Here, we propose a modification to the BayesR method that incorporates prior biological knowledge about which sites in the genome are more likely to affect the trait, using a flexible and practical approach. For instance, the biological knowledge can include lists of genes that are known to be important for trait expression, or specific genome sites that are likely to have functional consequences if mutated, such as non-synonymous coding sites. *A priori* we allocate all genotyped variants into classes, where each class of variants is believed to potentially differ in the probability that they contain causal variants for the trait. For example, one class could contain all non-synonymous coding variants within previously reported candidate genes such that this class may be enriched for causal variants compared to a random selection of variants. We call the method BayesRC. Previously Brondum et al. [6] proposed a modified BayesR approach (BayesRS) where prior estimates of the proportion of variance from different chromosome segments were used to weight the Bayesian priors for each segment. Our proposal differs because our prior is uniform across all variant classes such that the biological information will only influence the analysis if there is support for this in the data being analysed. The prior information is therefore more straightforward to incorporate in the model.

We evaluated our new method using data from dairy cattle where individuals had imputed genotypes for approximately two million variants in or near genome-wide coding regions as well as real or imputed high density SNP array genotypes. Due to the characteristically high LD (linkage disequilibrium) within dairy cattle breeds, we combined data from different geographical regions and breeds with the aim of reducing the longer distance LD to improve the precision of QTL (quantitative trait loci) discovery and prediction. We compare the accuracy of genomic prediction in validation individuals that are not closely related to the training individuals to more effectively determine the precision of QTL effect estimates.

Using simulated phenotypes as well as real milk production phenotypes, our results demonstrate several important advances:

1. Including imputed sequence variants from coding and regulatory regions increased the accuracy of genomic prediction compared to HD (high density) SNP array genotypes only, and enabled QTL detection among rare variants.
2. Our BayesRC method improved the power and precision of QTL discovery compared to BayesR.
3. BayesRC increased the accuracy of genomic predictions compared with the standard BayesR approach. The observed improvement was most apparent with increasing genetic distance between training and validation populations.

## Methods

Genomic prediction analysis was based on an imputed subset of sequence variants in dairy cattle with either simulated phenotypes or real milk production phenotypes. We generated three training (“reference”) data sets to test the new BayesRC method and compared these results with the BayesR method.

### Training and validation sets

The three training sets described below, are referred to as DANZ, AUS and AUS-Sim (summarised in Table 1). We employed several validation sets to represent different levels of relatedness to the training sets (see a principal components analysis of the genomic relationships in Additional file 1: Figure S1):

**Table 1:** Composition of three different mixed breed training (reference) sets, and several validation sets chosen to represent different levels of relatedness to the training sets

Training set: description	Training set: total	Training set: number per breed	Validation sets: in order of decreasing relatedness to the Training set
“DANZ” bulls of Dutch, Aust & N. Zealand origin with real genotypes and real phenotypes^a^	8920	7371 Holstein 1438 Jersey 111 Aust. Red	1. 869 Red Holstein bulls 2. 655 Australian Red cows
“AUS” Australian bulls & cows with real genotypes and real phenotypes^a^	16,214	11,527 Holstein: 3049 bulls, 8478 cows. 4687 Jersey: 770 bulls, 3917 cows.	1. 869 Red Holstein bulls 2. 655 Aust. Red cows
“AUS-Sim” Subset of above AUS set, with real genotypes and simulated phenotypes	10,314	7991 Holstein 2323 Jersey	1. 262 Holstein bulls only 2. 3940 Holstein bulls & cows 3. 869 Red Holstein bulls 4. 885 Aust. Red bulls & cows

^a^phenotypes were milk, protein and fat yield: in the case of bulls these are daughter averages from progeny test and all phenotypes were corrected for known fixed effects

1. **“DANZ**” – the training set included 8920 Dutch, Australian and New Zealand dairy bulls of pure-bred Holstein (black and white), Jersey and Australian Red breeds. The first validation set was made up of Red Holstein bulls. All sons or sires of this group were excluded from the training population. The second more genetically distant validation set was a group of Australian Red cows.
2. “**AUS**” - the training set included 16,214 Holstein and Jersey pure-bred bulls and cows of Australian origin (as described by Kemper et al [3]). The validation sets were the same as for the DANZ analysis (1. above).
3. “**AUS-Sim**” - The training set comprised the oldest 10,314 Holstein and Jersey animals from the AUS set (2. above) based on a year of birth cut off. The youngest Holstein bull and cows were assigned to two validation sets: the first was 262 bulls that were very closely related to the training set, while the second included these bulls as well as 3678 cows representing more genetic diversity than the bull only set. The third less related validation was the Red Holstein bulls as used for DANZ and AUS. Finally, the fourth most genetically distant validation was Australian Red breed cows and bulls.

### Genotypes and biological priors

All AUS individuals and some of the DANZ bulls were directly genotyped for the Illumina BovineSNP50 chip [7]. The remaining DANZ bulls were imputed from ~ 15,000 SNP to the BovineSNP50 chip. All individuals were then either directly genotyped or had imputed genotypes for the Illumina 800 K BovineHD beadChip. Further details of DANZ genotyping are published in [8] and details for AUS are published in [3]. In addition to HD 800 K SNP genotypes, we identified approximately two million sequence variants (SNP and indels) in gene coding regions and including variants 5000 bp up- and down-stream of these genes (based on annotation available for the reference bovine genome University of Maryland UMD3.1 assembly [9]). The discovery of sequence variants across these regions was carried out in Run 3.0 of the 1000 Bull Genomes project [10]. Beagle version 3 [11] was used to impute these sequence variants in all animals. The reference sequences used for imputation were 136 Holstein and 27 Jersey bulls combined from the 1000 Bull Genomes project (Run 3.0). The combined HD SNP and imputed sequence variants brought the total number of genotypes per animal to 2,785,440.

All 2.785 M variants were then defined as belonging to one of three broad categories based on annotation of the reference genome UMD3.1 (details in Additional file 1: Table S1). The first category, comprised variants predicted to cause a non-synonymous coding change, referred to as “NSC”. The majority were missense variants, but this NSC category also included variants such as splice site, inframe indels, frame shift and stop gained/lost mutations. The second category included variants in regions that were predicted to have potential regulatory roles: loosely referred to as “REG”. The REG variants were mainly those within a 5000 bp region upstream and downstream of genes, or in three/five prime untranslated genic regions or were non-coding exon variants. All other variants were from the Illumina HD 800 K SNP array and were allocated to the third category, referred to here as “CHIP”: these were mainly intergenic, but included some intronic and synonymous coding variants.

We then combined all the AUS Holstein and Jersey genotypes and used this data set to pre-select a subset of the most informative sequence variants. First we excluded those with Minor Allele Frequency (MAF) < 0.0002 using PLINK software [12]. We then excluded any one of a pair of variants in complete LD (*r*^2^ genotypic correlation >0.999) across groups of 500 adjacent variants in sliding windows of 50 variants (using PLINK). LD pruning was carried out first independently within each variant group (NSC, REG and CHIP) and then any REG or CHIP variant in complete LD with an NSC variant was removed. Last, all CHIP variants in perfect LD with a REG variant were removed. The remaining 994,019 variants, henceforth referred to as “SEQ”, were used for the analysis and included 45,026 NSC variants, 578,734 REG variants and 370,259 CHIP variants.

We also generated a standard set of SNP chip genotypes for each animal based on the Illumina HD 800 K SNP array that were in common with the full set of imputed 2.785 M sequence variants (ie. prior to pruning). This provided a comparison of the accuracy of genomic prediction using a standard 800 K genotype array or the SEQ genotypes. In total there were 600,641 SNP genotypes in this HD SNP array set, henceforth referred to as the “800 K” genotypes.

### Phenotypes

#### AUS

These phenotypes have previously been described by Kemper at al [3]. Briefly, the AUS bull phenotypes were daughter trait deviations (DTD) extracted from the ADHIS (Australian Dairy Herd Improvement Scheme) database. DTD are generated from nationwide progeny test data collected on many bull daughters, and have been corrected for known fixed effects such as herd, year and season. The AUS cows phenotypes were TD (trait deviations - also extracted from the ADHIS database) based on their own lactation records (3 lactations on average) and corrected for known fixed effects. Traits analysed were Milk, Fat and Protein Yield. A limited number of analyses were also carried out for Protein and Fat Percent derived from the Yield phenotypes as described by Kemper at al [3].

#### DANZ

These phenotypes are a subset of those described in [8] (ie. excluding Livestock Improvement Corporation, LIC, bulls). Briefly, the majority of Holstein and Jersey DANZ bulls had international MACE (multiple trait across-country evaluation) breeding values that were converted to de-regressed proofs (“DRP”) on the Australian scale. A total of 313 training bulls as well as the Australian Red bulls and cows did not have international MACE breeding values, and their DTD or TD were used instead (as suggested by Haile-Mariam et al [8]). The variance of DRP phenotypes was scaled to match the within breed DTD variance using records from bulls with both DRP and DTD. Additionally, data type by breed was included as a fixed effect in the analytical model. Traits analysed were Milk, Fat and Protein Yield. There was an overlap of 3819 AUS bulls that were included in the DANZ set of 8930 bulls.

#### AUS-Sim

Phenotypes were simulated for each animal as a complex trait with 4000 additive QTL effects that were simulated onto real genotypes chosen from SEQ variants. QTL were simulated by sampling 3485, 500 and 15 effects from each of three normal distributions, with a zero mean and variances; 0.0001$\sigma$^2^_g_, 0.001$\sigma$^2^_g_ and 0.01$\sigma$^2^_g_, respectively, where $\sigma$^2^_g_ is the additive genetic variance. The genetic value of the *j*^*th*^ animal was calculated as:

$$ GeneticValu{e}_j={\displaystyle \sum_{i=1}^{4000}{x}_{ij}}{\alpha}_i $$

where *α*_*i*_ is the *i*^th^ QTL effect and *x*_*ij*_ represents the *i*^th^ genotype (coded 0, 1 or 2 for genotypes aa, Aa and AA) of animal *j*. An environmental effect for each animal was sampled from a normal distribution and was added to the genetic value to produce phenotypes with heritability (h^2^) = 0.6. This relatively high h^2^ was chosen to mimic the highly accurate progeny test phenotypes of dairy bulls. Additionally a breed effect sampled from *N*(10,1) was added to the phenotypic value of all Holstein animals.

Three traits were simulated to provide a range of genetic architectures, where the 4000 QTL effects were simulated on different sets of SEQ variants that were chosen as follows:

1. QTL were randomly selected variants in or within 50 Kb of 790 “Lactation” genes including: 500 NSC, 2828 REG and 672 CHIP variants. The Lactation genes were candidate genes for milk production because they showed differential expression in association with experiments that altered milk yield (Additional file 1).
2. QTL randomly simulated on 1200 NSC and 2800 REG variants in and around coding regions, and dispersed genome-wide.
3. QTL simulated on variants chosen uniformly at random genome-wide, including: 177 NSC, 2241 REG and 1582 CHIP variants.

Pedigree information was obtained for all phenotyped animals, with data for overseas animals obtained from Interbull and Australian animals from ADHIS.

### BayesR

BayesR analytical methodology was described by Erbe et al [4] with further detail and additions in Kemper et al [3]. Our implementation exactly followed that of Kemper at al [3]. Briefly, BayesR uses an MCMC approach to estimate variant effects which are modelled as a mixture distribution of four normal distributions including a null distribution, *N*(0, 0.0*σ*^*2*^_*g*_), and three others: N(0, 0. 0001*σ*^*2*^_*g*_), *N*(0, 0.001*σ*^*2*^_*g*_), *N*(0, 0.01*σ*^*2*^_*g*_), where *σ*^*2*^_*g*_ is the additive genetic variance for the trait. The first distribution accommodates the likelihood that many variants have no effect on the trait, thus reducing the complexity of the model. The model fitted to the training datasets was:

1 $$ \mathbf{y}=\mathbf{X}\mathbf{b}+\mathbf{Z}\mathbf{a}+\mathbf{W}\mathbf{v}+\mathbf{e}, $$

where:

- **y** = vector of phenotypes for cows and/or bulls (TD, DTD or DRP)
- **X** = design matrix allocating phenotypes to fixed effects,
- **b** = vector of fixed effect solutions, where fixed effects included overall mean, breed, and when appropriate, data type – DRP, DTD, TD – nested within breed,
- **Z** = design matrix allocating phenotypes to polygenic breeding values,
- **a** = vector of polygenic breeding values: distributed *N*(0, **A***σ*^*2*^_*a*_): **A** = numerator relationship matrix calculated from sire and dam pedigree records and *σ*^*2*^_*a*_ = additive genetic variance not explained by the variants,
- **W** = design matrix of variant genotypes, centred and standardized to have a unit variance following [13],
- **v** = vector of variant effects, distributed as a mixture of the four distributions as listed above,
- **e** = vector of residual errors, distributed *N*(0, **E***σ*^*2*^_*e*_): with *σ*^*2*^_*e*_ = error variance. **E** is a diagonal matrix constructed as *diag(1/w*_*j*_*)*, where *w*_*j*_ is a weighting coefficient based on the number of records available for each animal as described in [3], and following [14]. This accounts for the variable accuracy of trait phenotypes (heterogeneous error variance) which arises in dairy cattle because bull phenotypes were calculated from <100 to many thousands of daughter lactation records, and cow TD were based on their own records (between 1 to 6 lactation records per cow).

Variant effects were assumed to belong to one of four normal distributions: *d*_1_*, d*_2_, *d*_3_ and *d*_*4*_. As in [4], the prior distribution for the proportion of SNP in each of these four distributions *(P*_d1_, *P*_d2_, *P*_d3_ and *P*_d4_) was ***P*** ~ Dirichlet (***α***) where ***α*** = [1,1,1,1]. Each iteration this was updated by sampling:

$$ \boldsymbol{P}\sim \mathrm{Dirichlet}\left(\boldsymbol{\alpha} +\boldsymbol{\beta} \right), $$

where ***β*** was a vector with the number of variants in each of the four distributions as currently estimated from the data. Each iteration, ***P*** was used in updating the conditional posterior probability that variant *i* belongs distribution *d* (details in [3]).

Variants with MAF < 0.002 in each training set were excluded from the analysis. For all BayesR models and traits we implemented five replicate chains of the Gibbs sampler, each chain running for 40,000 iterations with 20,000 iterations discarded as burn-in. Final parameter estimates were derived from the means of the sampled effects in the post burn-in iterations, obtained separately for each of the five chains. BayesR analyses were carried out with SEQ genotypes as well as with the 800 K SNP chip genotypes.

### BayesRC method

BayesRC used the same approach as BayesR except that *a priori* independent biological information was used to allocate each variant to a specific “class” *c* (where *c* ≥ 2), where the purpose is to provide one or more classes that are enriched for QTL. For example, all variants in or close to candidate genes could be allocated to class I, while all other variants could be in class II. As for BayesR, the variant effects for members of class I are assumed to belong to a mixture of four normal distributions with proportions (*P*_*d*1_cI_, *P*_*d*2_cI_, *P*_*d*3_cI_, *P*_*d*4_cI_,) while the variant effects that are members of class II belong to an independent mixture of the four distributions with proportions (*P*_*d*1_cII_, *P*_*d*2_cII_, *P*_*d*3_cII_, *P*_*d*4_cII_,), etc. In BayesRC a small modification in the BayesR algorithm allows updating of the distribution of QTL effects within classes: an advantage if a particular class is enriched for QTL. Within each class *c*, we used a uniform Dirichlet prior (as in BayesR) for the proportion of effects in each distribution: ***P***_***c***_ ~ Dir(***α***_***c***_), where ***α***_***c***_ **=** [1,1,1,1]. This was updated each iteration within each class:

$$ {\boldsymbol{P}}_{\boldsymbol{c}}\sim \mathrm{D}\mathrm{i}\mathrm{r}\left({\boldsymbol{\alpha}}_{\boldsymbol{c}}+{\boldsymbol{\beta}}_{\boldsymbol{c}}\right), $$

where ***β***_***c***_ was the current number of variants in each of the four distributions within class *c*, as estimated from the data. Thus, we used a relatively uninformative prior for all classes, but within a class the posterior proportion of variants in each distribution was informed by the data and could vary from one class to the next. If a class is found to be enriched for QTL this increases the probability that a true QTL effect in this class will be included in the model. The prior of ***α***_***c***_ ***=*** **1** can be argued to have little influence on the posterior distribution provided that there is a reasonably large number of variants per class. The updating of all other parameters was carried out as described for BayesR [3].

We consider three versions of BayesRC (BayesRC Seq, BayesRC Lact and BayesRC Rlact) defined by how the prior allocated SNP to one of three classes, as described in Table 2. In BayesRC Seq the variant categories in SEQ genotypes (NSC, REG and CHIP) provided a simple biological prior, under the hypothesis that NSC should be most enriched for causal variants, REG somewhat enriched and CHIP least likely to contain causal variants. In BayesRC Lact, the prior was based on a set of 790 candidate genes associated with milk production (referred to as “Lact” genes: Additional file 2) that had been discovered in an independent microarray gene expression study [15] (see Additional file 1). Although the DGAT1 (diacylglycerol O-acyltransferase homolog 1) gene was not included in the original microarray experiment, we added it to the Lact set because a causal mutation in this gene has been demonstrated to have a very large effect on fat, milk and protein yield [16]. In the third version, BayesRC Rlact, we used the same prior as BayesRC Lact except that we replaced the Lact gene set with a randomly generated set of 790 genes to provide a null model.

**Table 2:** Description of BayesRC models used to analyse the SEQ ^a^ genotype data

Name of BayesRC Model	Variant Allocation to Classes I, II and III	Number of variants per class^c^
BayesRC Seq	I. NSC (non-synonymous coding) II. REG (potentially regulatory) III. CHIP (HD SNP chip variants)	45,026 578,734 370,259
BayesRC Lact	I. NSC & in Lact ^b^ genes II. All variants other than NSC that overlap Lact gene regions (±50Kb) III. All other SEQ variants not in class I or II	4650 64,518 924,851
BayesRC RLact	I. NSC & in random set of 790 genes II. Variants other than NSC that overlap a random set of 790 genes (±50Kb) III. All other variants not in class I or II	4350 61,748 927,921

^a^SEQ = pruned set of 994,019 genome-wide sequence variants from coding and regulatory regions as well as SNP from a high density genotyping array. Variants were allocated to one of three BayesRC classes as listed

^b^Lact refers to a set of 790 candidate genes shown in an independent study to be differentially expressed in association with altered milk production

^c^Numbers generally reduced slightly from those listed because variants with MAF < 0.002 in any given training population were also excluded from the analyses

### Genome-wide association analysis - GWAS

An association study was conducted in the AUS dataset using ‘SNP Snappy’ [17]. This process fitted a model similar to Eq. 1, but replaced the term for all SNP genotypes (**Wv**) with a single SNP regression of phenotype on genotype, one SNP at a time. That is, as well as the SNP regression, the model included the overall mean, fixed effects, a polygenic term and phenotypes were weighted for heterogeneous error variance [14].

### GBLUP

A traditional GBLUP method was implemented for the simulated data as described in [3] using ASReml software [18] and fitting the model described in Eq. 1. As for BayesR, all variants are fitted in the model simultaneously, but GBLUP linear mixed model assumes each variant has an effect sampled from the same normal distribution.

### Accuracy of genomic prediction

The accuracy of genomic prediction was estimated from the correlation between the predicted genetic value $\left({\widehat{\mathbf{y}}}_{\mathbf{v}}=\mathbf{W}\widehat{\mathbf{v}}\right)$ and the phenotypes (TD, DTD or DRP) for all validation sets. For consistency, the residual polygenic value was not included in the prediction of genetic value because some validation sets were not connected through the pedigree with the training population. In the AUS-Sim data we used the same approach but the accuracy was measured by the correlation between the predicted genetic value (**ŷ**_**v**_) and the simulated true genetic value. In AUS-Sim we assessed the bias of the predictions using the regression coefficient of the true genetic value on the predicted genetic value. Accuracies and regression coefficients were calculated within each of five MCMC chains and the reported value is the mean.

## Results

### Genotype LD and MAF

The allele frequency spectrum of the 994,019 imputed SEQ variants was similar for all three cattle breeds used in this study (Holstein, Jersey and Australian Red). A larger proportion of NSC and REG variants had MAF < 0.1 (55 % and 49 % respectively) compared to CHIP variants (21 %) (Additional file 1: Figure S2). The proportion of all polymorphic loci not segregating across both the Holstein and Jersey breeds was; 24 % for NSC, 19 % for REG and 4 % for CHIP variants. The LD among CHIP variants was on average higher than LD between NSC and CHIP variants (Additional file 1: Table S2).

### Simulated phenotypes – accuracy of genomic prediction

The AUS-Sim data (Table 1) used real genotypes with three different simulated trait phenotypes (each with 4000 QTL). For each trait we analysed the data with GBLUP, BayesR and three versions of BayesRC (BayesRC Seq, BayesRC Lact and BayesRC Rlact) that differed in the biological priors used to allocate variants to one of three classes (Table 2). The simulated traits were developed to test specific BayesRC priors:

1. QTL simulated on variants in or close to the 790 Lact genes. The BayesRC Lact was the most appropriate model for this trait because QTL were allocated either to class I or II. The QTL represented 13 % of all class I variants and 6 % of all class II variants: that is there was enrichment for QTL, particularly in class I.
2. QTL simulated on 1200 NSC and 2800 REG variants, randomly chosen genome-wide. The BayesRC Seq model was the most appropriate for this trait because all QTL were allocated to class I (NSC) and class II (REG). However, the QTL represented only 3 % of class I variants and 0.5 % of all class II variants: that is enrichment for QTL in these two classes was weak.
3. QTL simulated on random variants genome-wide, including NSC, REG and CHIP variants. This trait represents a null model with QTL randomly dispersed across all classes, therefore none of the BayesRC priors were biologically informative: that is there was no class enrichment for QTL.

**Fig. 1:**
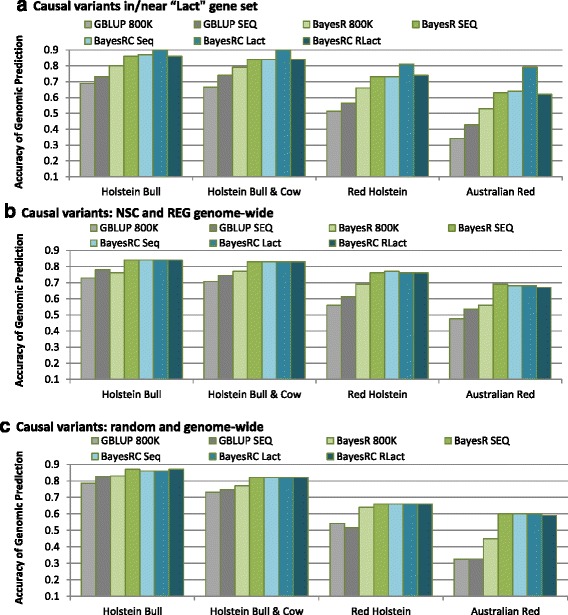
**a**, **b** and **c** Accuracy of genomic prediction for real genotypes with simulated phenotypes (3 traits with h^2^ = 0.6) with a range of BayesR and BayesRC models (AUS-Sim data). BayesR models used 800 K SNP array genotypes or sequence data (SEQ), while all BayesRC models used SEQ data (models described in Table 2). The results are shown for the three simulated traits: **a** QTL simulated on variants in or close to a set of 790 Lact genes, **b** QTL simulated on NSC or REG variants only and **c** QTL simulated at random genome-wide on NSC, REG and CHIP variants

Figure 1 compares the accuracy of genomic prediction estimated as the correlation between predicted genetic values and true genetic values. In all comparisons the accuracy of GBLUP was lower than BayesR and BayesRC. For all traits, the accuracy of prediction decreased with decreasing relatedness between training and validation sets (Fig. 1). However, this decrease was generally the more severe with GBLUP compared to BayesR or BayesRC. As expected, the accuracy of prediction generally increased using the SEQ genotypes, in which causal variants were present, compared to using 800 K variants (no QTL present). For BayesR, the relative gain from SEQ variants increased dramatically in the least related Australian Red validation (Fig. 1), indicating improved precision of estimated QTL effects. Smaller differences were observed for GBLUP because the GBLUP model fits a quasi-infinitesimal model with all effects estimated from a single normal distribution, resulting in the effect of a single QTL being spread across many variants in moderate LD with the QTL. BayesR on the other hand is better at predicting more precise effects, and can more accurately estimate the larger QTL effects because the QTL effects are modelled as a mixture distribution [3, 5].

For Trait 1, accuracy was highest for the BayesRC Lact model (Fig. 1a) where class I and II were enriched for true QTL. Importantly, the accuracy of the BayseRC Lact model persisted in the more genetically distant validation sets indicating that QTL effects were estimated more precisely. For example, in the Australian Red breed the BayesRC Lact accuracy was 16 % higher than the BayesR SEQ model and was almost as high as accuracy in the Red Holsteins. We also tested models equivalent to the BayesRC Lact, but with only two thirds or one half of the Lact genes correctly identified, thus one third or one half of QTL were mis-allocated to class III (Additional file 1: Table S3 - BayesRC 2/3Lact and BayesRC 1/2Lact). Although these latter models represented much less informative biological priors (ie. reduced enrichment of QTL in class I and II compared to BayesRC Lact) they still conferred an advantage in accuracy for Trait 1 compared to BayesR (Additional file 1: Table S3). Again, this was most apparent for the Australian Red validation (9 % and 6 % improvement).

For Trait 2, although all QTL were contained in classes I and II of the BayesRC Seq model, this did not lead to an increase in accuracy, probably because the QTL represented only 3 % and 0.5 % of all variants in the two classes respectively. That is, enrichment for QTL in these classes was too low. For the BayesRC RLact (random allocation of QTL to classes) and all BayesRC models tested on Trait 3 (no enrichment of QTL in classes I or II) there was no difference in accuracy compared to the BayesR SEQ model (Fig. 1). Importantly, this indicates that there was no penalty for uninformative class specification.

### Simulated phenotypes – genetic architecture

The genetic architecture of the simulated traits was relatively accurately recovered in BayesR and BayesRC models with SEQ genotypes. For instance, in Trait 1 the proportions of QTL in each of the four distributions within each class of the BayesRC Lact model approximated the true proportions (Table 3). Although no causal variants were allocated to class III, a small number of QTL were estimated to be present in this class probably because some variants just outside the Lact gene regions were in high LD with Lact gene variants. (See also Additional file 1: Table S4).

**Table 3:** Average number of QTL estimated per distribution and per class of the BayesRC Lact model^a^, compared with the true number of simulated QTL

CLASS		Number of QTL per Distribution			Total per Class
		N(0,0.0001$\sigma$ ^2^_g_)	N(0,0.001$\sigma$ ^2^_g_)	N(0,0.01$\sigma$ ^2^_g_)	
Class I	TRUE Number	436	63	1	500
	BayesRC Lact	444	36	4	484
Class II	TRUE Number	3049	437	14	3500
	BayesRC Lact	2512	346	16	2874
Class III	TRUE Number	0	0	0	0
	BayesRC Lact	219	11	1	231
Total per distribution	TRUE Number	3485	500	15	
	BayesRC Lact	3175	393	21	

^a^ Results are for Trait 1 (AUS-Sim data) where QTL were simulated in Lact gene regions only

### Simulated phenotypes – QTL discovery

**Fig. 2:**
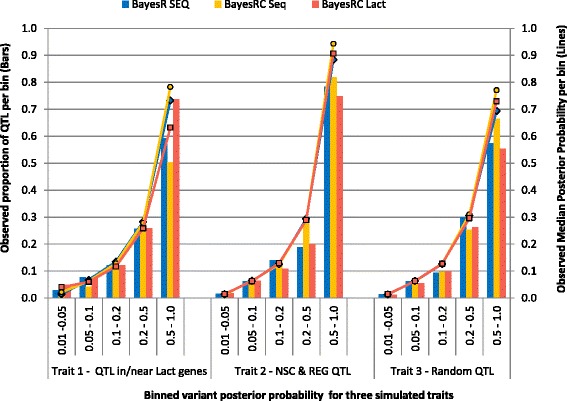
The observed proportion of true QTL among variants with posterior probabilities falling in one of five bins (bars) compared to the median posterior probability for variants in each bin (lines). Posterior probabilities are calculated as the proportion of iterations that a variant was estimated to have a real effect on the trait. Results are from the AUS-Sim data (real cattle genotypes with 4000 simulated QTL) for three simulated traits with BayesR SEQ, BayesRC Seq and BayesRC Lact models (see Table 2 for description of BayesRC models)

In the Bayesian framework, the observed posterior probability of a variant having a non-zero effect should provide a direct measure of the relative likelihood that a variant is causal or is in very high LD with a real QTL. For all three simulated traits, the posterior probability generally reflected close to the true probability that a variant was a QTL (Fig. 2). That is, if 100 variants with a posterior probability > 0.25 were selected as potential causal variants, then at least 25 were real QTL. This confirms that the posterior probability statistic is generally well calibrated and could be used to make informed decisions on selecting variants for further study. The appropriate choice of posterior probability threshold for selection of variants would depend on the particular study objective. For studies designed to confirm causal mutations, it would be wise to choose a small number of variants with a high posterior probability and with consideration of other informative biological data. Alternatively if the objective is to find a subset of informative SEQ variants to include on a custom array for genomic prediction, then the appropriate threshold would be considerably lower.

**Fig. 3:**
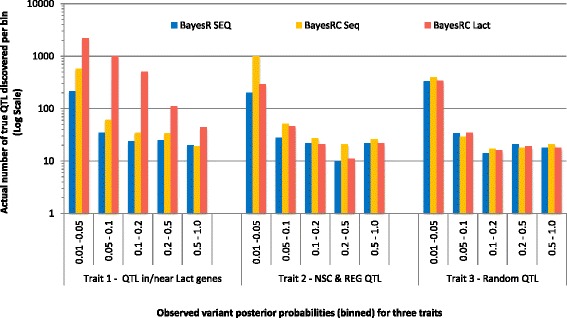
Number of true QTL discovered (log scale) within groups of variants binned on posterior probabilities, for three simulated traits. The sum across all bins is the number of true QTL with posterior probability > 0.01 out of a total of 4000 simulated QTL. Results are shown for the AUS-Sim data (real genotypes with 4000 simulated QTL) applying a range of BayesR and BayesRC models (see Table 2 for description of BayesRC models). Posterior probabilities are calculated as the proportion of iterations that a variant was estimated to have a real effect on the trait

The power to detect the 4000 QTL from approximately 900,000 variants was highest for Trait 1 with the BayesRC Lact model (Fig. 3). For example, in BayesRC Lact, 115 simulated QTL were recovered with a posterior probability > 0.25 (667 with posterior probability > 0.1), while other analyses recovered less than 40 (89) QTL above this 0.25 (0.01) threshold. For Trait 2, BayesRC Seq identified 42 QTL with posterior probability ≥ 0.25 (74 with posterior probability ≥ 0.1) compared to less than 30 (56) in other models. Therefore, although the BayesRC Seq did not improve prediction accuracy because class enrichment for QTL was weak, it did provide a small advantage for QTL discovery (Fig. 3). For Trait 3, as expected, the number of QTL detected was similar across BayesR and all BayesRC models because no class was enriched for QTL (Fig. 3).

### Real phenotypes – accuracy of genomic prediction

In the DANZ analysis, there was generally a consistent trend for accuracy of prediction to increase with variant density moving from 800 K to SEQ (Table 4). Overall, the accuracy of prediction in the Australian Red cow validation set was very low because cow phenotypes were less reliable as an indicator of true genetic value than bull phenotypes which were based on a progeny test. This is in contrast to the simulated data where bulls and cows had equally reliable data and also accuracy was measured as the correlation between the predicted genetic value and true genetic value.

**Table 4:** Accuracy^a^ of the DANZ training predictions for Fat, Milk and Protein Yield in the Red Holstein bull and the Australian Red cow validation sets

	FAT		MILK		PROTEIN	
Analytical Model^b^	Red Hol	Aust Red	Red Hol	Aust Red	Red Hol	Aust Red
BayesR 800 K	0.565 (0.001)	0.344 (0.003)	0.650 (0.001)	0.317 (0.003)	0.603 (0.001)	0.200 (0.001)
BayesR SEQ	0.572 (0.001)	**0.354** (0.004)	0.663 (0.002)	0.308 (0.005)	0.612 (0.001)	0.220 (0.003)
BayesRC Lact	**0.576** (0.002)	0.353 (0.002)	**0.664** (0.001)	**0.325** (0.004)	**0.616** (0.001)	**0.226** (0.003)
BayesRC RLact	0.571 (0.001)	0.352 (0.002)	0.657 (0.001)	0.302 (0.005)	0.612 (0.001)	0.218 (0.002)

^a^Estimated as the average correlation between the genomic prediction and corrected phenotypes. The highest accuracy is in bold font in each column. Numbers in in brackets indicate relative convergence of 5 independent Bayesian MCMC chains (estimated from [SD of the mean accuracy]/√5). Note: the numbers in brackets should not be interpreted as a “standard error” because they are estimated from 5 Bayesian MCMC chains run on the same data set

^b^BayesR models used either 800 K SNP array (600,640 genotypes) or 994,019 sequence variants (SEQ). The BayesRC model definitions are given in Table 2

For the DANZ analysis, there was a trend for slightly increased accuracy with the BayesRC Lact model compared to the BayesR SEQ model in both validation sets except in the Australian Red validation for Fat Yield. The Lact genes were expected to be most highly associated with Milk Yield because of the experimental design used to identify these genes (Additional file 1). However increased Milk Yield is often associated with an increase in Protein and Fat Yield. Overall, the accuracy of BayesRC RLact (variant classes based on a random gene set instead of Lact genes) were similar to BayesR SEQ and slightly lower than BayesRC Lact accuracy.

In the AUS data, the accuracy of genomic prediction showed similar trends to those in DANZ data: increased accuracy with BayesR SEQ compared BayesR 800 K and slightly higher accuracy with BayesRC Lact for Milk Yield (Table 5). In the Australian Red validation the accuracies for Protein Yield were very low indicating that these validation results are less reliable, so it is not surprising that these results did not follow a clear trend.

**Table 5:** Accuracy^a^ of the AUS training predictions for Fat, Milk and Protein Yield in the Red Holstein bull and Australian Red cow validation sets

	Fat Yield		Milk Yield		Protein Yield	
Analytical Model^b^	Red Hol	Aust Red	Red Hol	Aust Red	Red Hol	Aust Red
BayesR 800 K	0.527 (0.002)	0.265 (0.001)	0.580 (0.001)	0.235 (0.005)	0.530 (0.002)	0.155 (0.004)
BayesR SEQ	**0.543** (0.001)	0.275 (0.002)	0.601 (0.004)	0.258 (0.008)	0.548 (0.002)	0.174 (0.005)
BayesRC Lact	0.540 (0.003)	**0.281** (0.004)	**0.604** (0.002)	**0.278** (0.012)	**0.554** (0.002)	0.154 (0.015)
BayesRC RLact	0.541 (0.002)	0.272 (0.004)	0.602 (0.004)	0.253 (0.012)	0.551 (0.002)	**0.180** (0.006)

^a^Estimated as the correlation between the predicted genomic values and corrected phenotypes. The highest accuracy is in bold font in each column. Numbers in in brackets indicate relative convergence of 5 independent Bayesian MCMC chains (estimated from [SD of the mean accuracy]/√5). Note: the numbers in brackets should not be interpreted as a “standard error” because they are estimated from 5 Bayesian MCMC chains run on the same data set

^**b**^BayesR models used either 800 K SNP array (600,640 genotypes) or 994,019 sequence variants (SEQ). The BayesRC model definitions are given in Table 2

### Real phenotypes – genetic architecture

The average number of variant effects estimated per non-zero variance distribution (variance of 0.0001$\sigma$^2^_g_, 0.001$\sigma$^2^_g_, and 0.01$\sigma$^2^_g_) were similar for all models with SEQ data (results for BayesR SEQ and BayesRC Lact shown in Table 6). The overall number of variants estimated per trait was higher than in our simulation with most in the smallest variance distribution.

**Table 6:** Average number of variant effects per non-zero distribution (variances 0.0001$\sigma$ ^2^_g_, 0.001$\sigma$ ^2^_g_, and 0.01$\sigma$ ^2^_g_) of BayesR SEQ and BayesRC Lact models^a^

Trait	Model	Number of Variant Effects per Distribution					
		N(0,0.0001$\sigma$ ^2^_g_)		N(0,0.001$\sigma$ ^2^_g_)		N(0,0.01$\sigma$ ^2^_g_)	
		AUS	DANZ	AUS	DANZ	AUS	DANZ
Milk Yield	BayesR SEQ	4263	5239	60	91	7	9
	BayesRC Lact	4276	5294	56	89	9	11
Fat Yield	BayesR SEQ	4769	5969	14	28	5	8
	BayesRC Lact	4774	5841	24	43	7	10
Protein Yield	BayesR SEQ	4604	6292	40	38	5	6
	BayesRC Lact	4641	6292	39	41	7	8

^a^ Results are for Milk, Fat and Protein Yield in both the DANZ and AUS training sets

In the BayesRC Lact analysis of Milk Yield, class I and II variants appeared to be enriched for QTL effects (Table 7). For instance, in the AUS dataset, 3.9 % of class I variants were sampled in the 0.0001$\sigma$^2^_g_ distribution whereas in BayesR SEQ, only 0.86 % of all variants were in this distribution. The highest fold enrichment was in Class I for SNP effect distributions with 0.001$\sigma$^2^_g_ and 0.01$\sigma$^2^_g_ variance (Table 7).

**Table 7:** Proportion of non-zero variant effects estimated per distribution, within each class of the BayesRC Lact model for Milk Yield

Model	Class	Number of Variants	Proportion of Variant Effects per Distribution					
			N(0,0.0001$\sigma$ ^2^_g_)		N(0,0.001$\sigma$ ^2^_g_)		N(0,0.01$\sigma$ ^2^_g_)	
			AUS	DANZ	AUS	DANZ	AUS	DANZ
BayesR SEQ	N/A	**909,143**	**0.86 %**	**0.58 %**	**0.01 %**	**0.01 %**	**0.002 %**	**0.001 %**
BayesRC Lact	Class I	3709	3.91 %	3.76 %	0.38 %	0.24 %	0.07 %	0.045 %
BayesRC Lact	Class II	57,541	1.01 %	0.65 %	0.03 %	0.04 %	0.004 %	0.006 %
BayesRC Lact	Class III	847,892	0.43 %	0.57 %	0.01 %	0.007 %	0.0003 %	0.0007 %

Results are given for both AUS and DANZ training sets, and are compared to the distribution of variant effects in the BayesR SEQ model (bold figures)

To confirm that class I and II variants in BayesRC Lact were enriched for milk yield QTL we tested their ability to predict phenotype compared with the same number of randomly chosen variants. We derived a separate prediction equation for each class (I, II and III) using the variant effects estimated from BayesRC Lact (DANZ), for each of the five replicated MCMC chains. We then randomly selected 790 gene regions and allocated equivalent numbers of SEQ variants to class I, II and III as in BayesRC Lact. Prediction equations for these random variant sets were derived from the BayesR SEQ estimated variant effects. This was replicated 10 times by sampling a new set of 790 genes with replacement, giving a total of 50 replicates (because prediction equations were derived for each of the five BayesR SEQ chains). The accuracy of all prediction equations for each class was estimated in the Red Holstein validation set and averaged across replicates. We repeated the same procedure for Fat and Protein Yield.

**Fig. 4:**
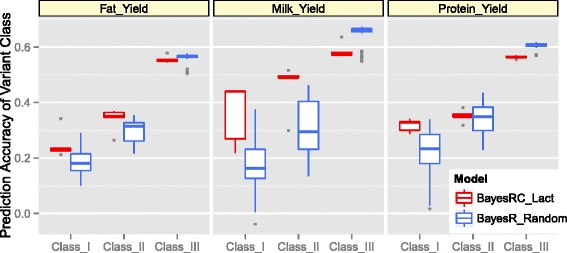
Accuracy of prediction (real DANZ data) per variant class of the BayesRC Lact model compared with BayesR predictions using a matching number of randomly selected variants (BayesR_Random). Accuracy was estimated as the correlation between the predicted value and the Red Holstein phenotypes (for Fat, Milk and Protein Yield). The boxplot shows the median and range of values for all replicates (grey dots representing outliers)

For Milk Yield there was higher prediction accuracy from BayesRC Lact class I and II equations compared to those from the random gene classes I and II, confirming enrichment of Milk Yield QTL in class I and II (Fig. 4). For Protein Yield the BayesRC Lact accuracies confirmed some QTL enrichment in class I only. The accuracies for Fat Yield suggested a low level of QTL enrichment in class I and II but somewhat less than observed for Milk Yield. Enrichment for Milk and Protein Yield QTL was further substantiated by the accuracy of class III being lower for BayesRC Lact than that of the random predictions (Fig. 4) suggesting some depletion of QTL in Class III.

### Real phenotypes – QTL discovery

Use of SEQ compared to CHIP genotypes was expected to improve QTL discovery, particularly if a causal variant was rare and/or present in the SEQ data. We detected a number of strong QTL signals in the SEQ analyses in regions where no QTL were detected in the 800 K analyses (ie. variants with a posterior probability > 0.25 of being a QTL effect). One such example was a rare REG variant (MAF < 0.01 in Holstein and not segregating in Jersey animals) that lies 2777 bp upstream of the SMEK1 (suppressor of mek1) gene coding region (Additional file 1: Figure S3). A second example is a rare variant (MAF of 0.02 in Holstein and 0.002 in Jersey) 4949 bp upstream of the CSH2 (chorionic somatomammotropin hormone 2) gene (Additional file 1: Figure S4).

**Fig. 5:**
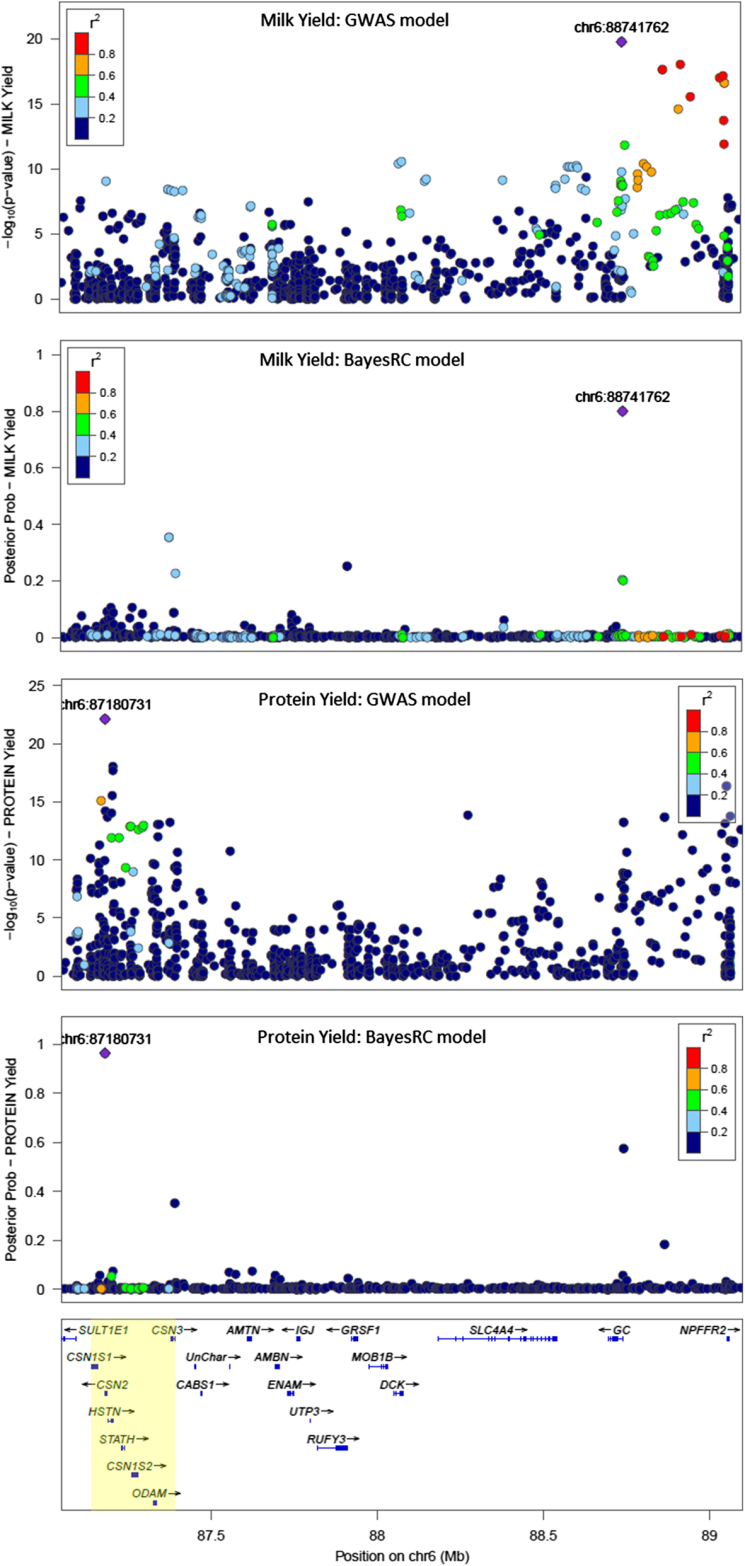
QTL discovery with GWAS (-log_10_ of *p*-value) and BayesRC Lact (posterior probability) for Milk and Protein Yield around the casein gene cluster (yellow highlight) and GC gene. The BayesRC variant with the top probability (real AUS data) is shown by a purple diamond in each plot (labelled with chromosome and bp position). The strength of LD (r^2^) between this top variant and all others is colour coded

Testing one SNP at a time is the most common method of QTL analysis in genome wide association studies (GWAS). Therefore we compared the power and precision of QTL discovery using single SNP regression (“GWAS”), BayesR and BayesRC in several previously documented candidate gene regions. Figure 5 compares QTL discovery with both GWAS and the BayesRC Lact model for Protein and Milk Yield in and around the casein gene cluster (CSN1S1, CSN2, CSN1S2, CSN3: caseins account for a large proportion of milk protein). The GWAS results showed many strong signals across the casein cluster, while the BayesRC Lact model suggested there may potentially be two causal variants for Protein Yield: one associated with beta-casein gene (CSN2) and the other with kappa-casein (CSN3). This highlights the ability of the Bayesian model to differentiate just one or two most probable variants compared to the GWAS approach which finds many variants in an extended region with high –log_10_*p*-values. There were many variants in medium to strong LD with the top BayesRC variants at 87,180,731 and 88,741,762 (Fig. 5). In the GWAS analysis of Protein Yield it is unclear whether the high –log_10_*p*-values around the GC (group-specific component, vitamin D binding) gene arise due to LD with one or more causal variants in the nearby casein gene cluster. However, the BayesRC Protein Yield analysis indicates good evidence for an additional causal mutation near the GC gene because the most probable variant in this region is not in strong LD with the highest probability variant in the Casein cluster (Fig. 5). Furthermore, the same candidate variant close to the GC gene (88,741,762 bp) also had the highest BayesRC posterior probability in this region for Milk Yield (Fig. 5). Thus the Bayes RC analysis suggests three causal variants in this region: two near the casein genes mainly affecting Protein Yield and one near the GC gene affecting Milk and Protein yield.

**Fig. 6:**
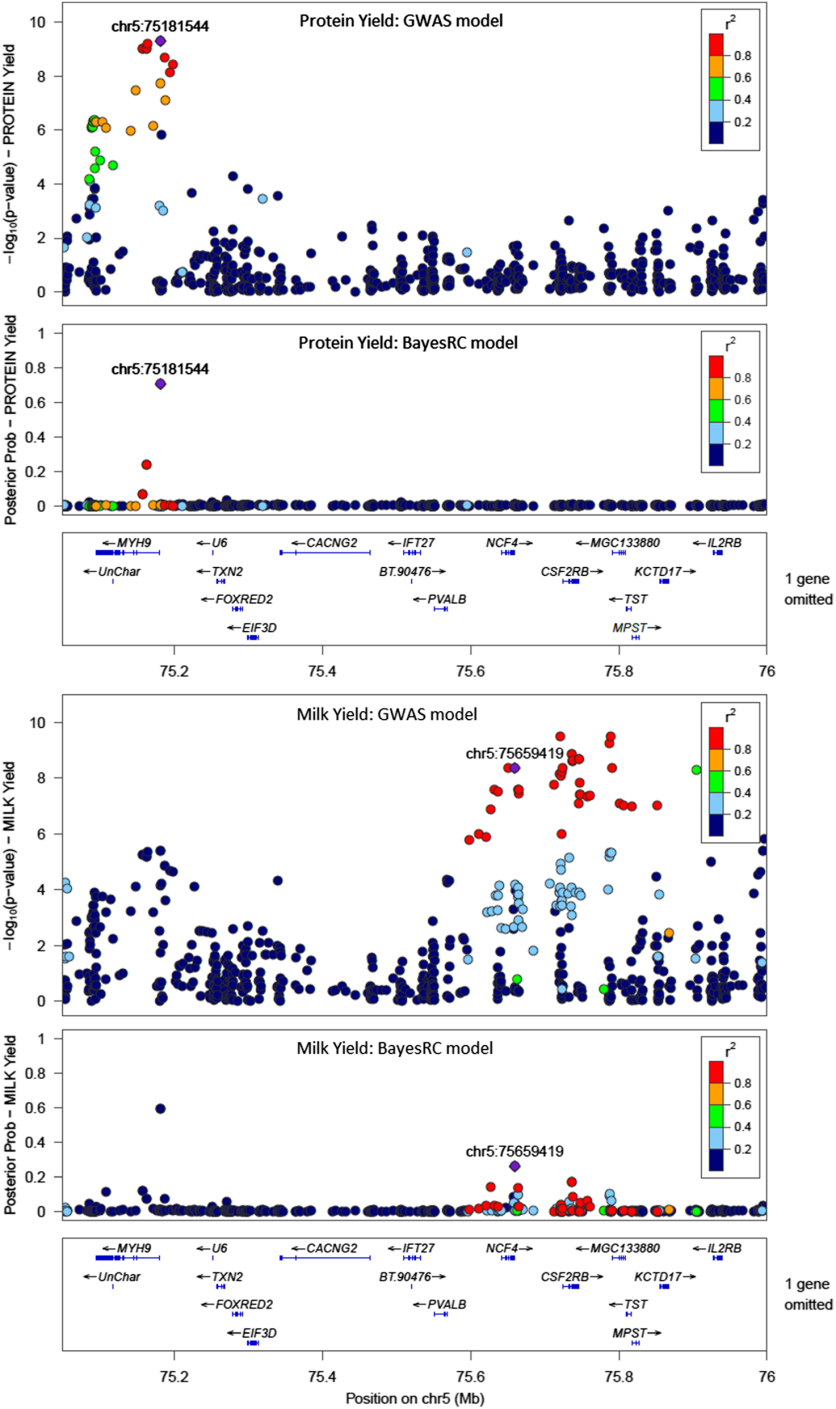
QTL discovery with GWAS (-log *p*-value) and BayesRC Lact (posterior probability) for Milk and Protein Yield across a 1 Mb region of Chromosome 5. The BayesRC variant with the top posterior probability in a given region (real AUS data) is shown by a purple diamond (labelled with chromosome and bp position). The LD (r^2^) between this variant and all others is colour coded

A second comparison of GWAS and BayesRC Lact is given in Fig. 6 for a region on Chromosome 5 which again showed strong associations with Milk and Protein Yield. In the GWAS Protein analysis it is difficult to determine the number of QTL, while in the BayesRC analysis the evidence is more compelling that there are at least two QTL regions. The high probability variant at 75.18 Mb lies just 1635 bp downstream of the MYH9 (non-muscle myosin, heavy chain 9) gene and affects both Milk and Protein Yield. There is evidence of another QTL region around the NCF4 (neutrophil cytosolic factor 4) and CSF2RB (colony stimulating factor 2 receptor beta common subunit) genes (from 75.6 to 75.9 Mb) affecting only Milk Yield. The BayesRC analysis shows several small peaks of posterior probabilities possibly indicating that, due to the very strong LD across this region, the analysis cannot determine which SNP or gene is most likely to be causal.

**Fig. 7:**
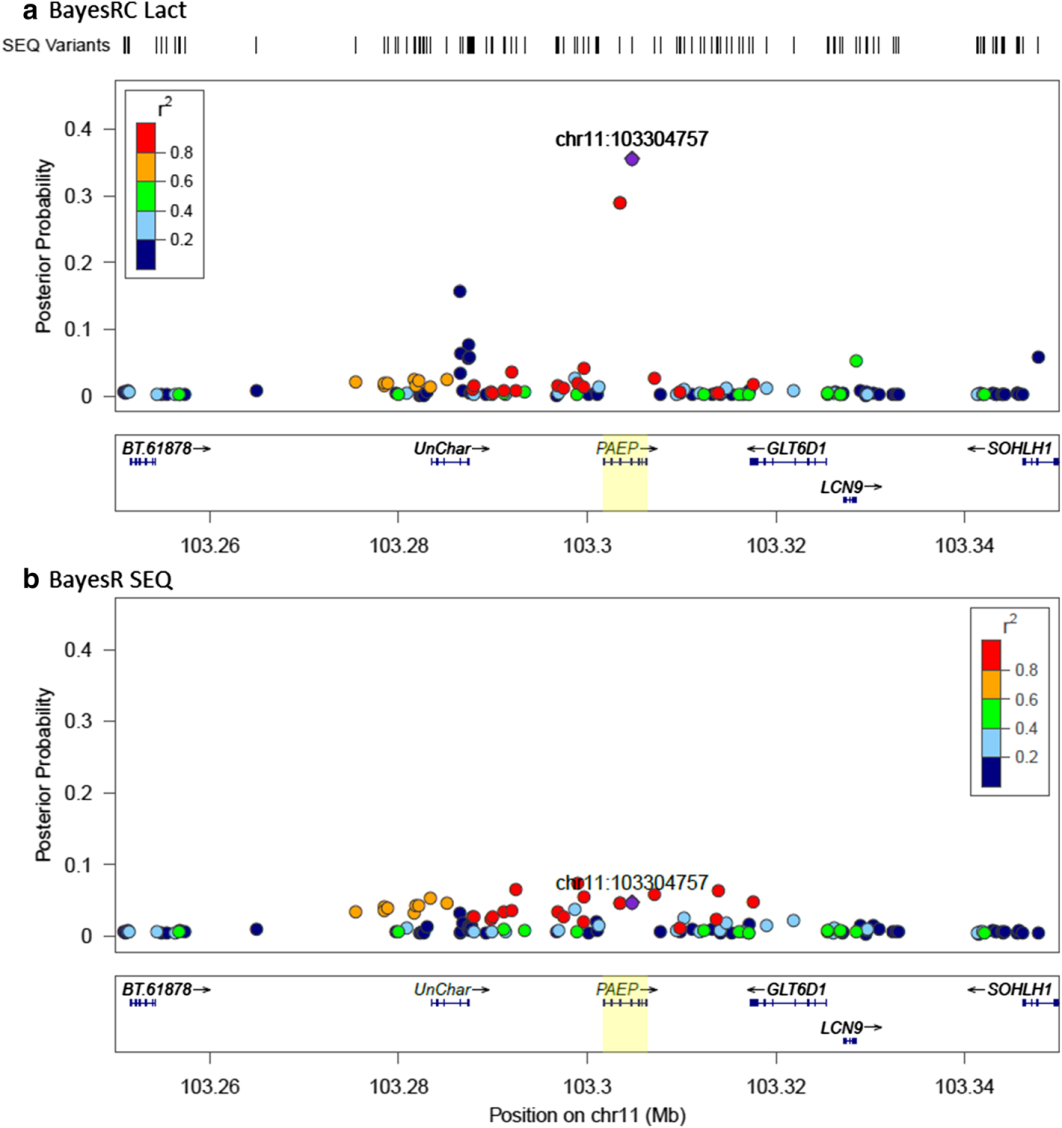
**a** and **b**. QTL discovery: posterior probabilities of variants in the PAEP gene region for BayesRC Lact (**a**) and BayesR SEQ analysis (**b**). The BayesRC Lact variant with the top posterior probability (real DANZ data) is shown by a purple diamond in each plot (labelled with chromosome and bp position) and the LD (r^2^) between this variant and all others is colour coded. The position of the SEQ variants fitted in the model is also shown above

We also found evidence of improved power of QTL discovery in BayesRC Lact compared to the BayesR SEQ model in a number of QTL regions. An example of this is provided by the PAEP gene (alias LGB, beta lactoglobulin) that was included in our Lact gene set. This is an important milk whey protein and mutations in and close to this gene have previously been shown to be associated with milk protein traits [19–21]. Figure 7 compares the posterior probabilities of variants in this region for BayesR SEQ and BayesRC Lact analysis and also shows LD between the highest posterior probability variant (BayesRC) and all other variants in the region. A single variant (103,304,757 bp) stands out with a very high BayesRC posterior probability for Protein Yield (Fig. 7a) as well as one other adjacent variant at 103,303,475 (both these variants were also the most significant in the GWAS). In contrast, the BayesR posterior probability is lower and spread across several variants all in strong LD over a 50Kb segment (Fig. 7b). Also of note in Fig. 7a is a small peak of higher posterior probability variants over a gene labelled as uncharacterised (“UnChar”) that are not in LD with those around PAEP. This uncharacterised gene was not included in the Lact gene set but is now annotated on the NCBI (National Center for Biotechnology Information) “gene” repository (http://www.ncbi.nlm.nih.gov/gene/) as a duplicated PAEP-like protein coding gene (RefSeq status “MODEL”).

Table 8 provides a short list of candidate genes (with full gene names provided in Table 9) identified by variants in or close to genes (within 5000 bp) that showed the strongest evidence for associations with one or several traits (AUS data). All variants listed had a posterior probability > 0.25 in the BayesRC Lact analysis and there was additional evidence in support of the candidate genes listed: they were either validated in the DANZ analysis, were associated with more than one milk trait (including milk fat and milk protein percent), were in the Lact gene set and/or were positively differentially expressed in lactating mammary tissue compared to 17 other tissues of a lactating dairy cow [22].

**Table 8:** Candidate genes identified by listed variants in coding or regulatory regions with a posterior probability ≥ 0.25 for Milk, Protein or Fat Yield (AUS BayesRC Lact)

Gene_ID (see names in Table 9)	DE^a^	Milk Y	Prot.Y	Fat Y	P%	F%	Evidence^b^	Variant type (distance from gene or SIFT prediction)	Variant position (chrom : bp)
ROBO1	*n*		+	+			P	upstream (1823 bp)	1:26212317
SLC37A1	*++*	+					L,D	downstream (4005 bp)	1:144441230
PSMB2	*n*	-		-			P,L	missense (SIFT:deleterious)	3:110752811
OGDH	*n*	+	+				P	downstream (4105 bp)	4:77454411
MYH9	*n*	+	+				P,L	upstream (1635 bp)	5:75181544
NCF4	*n*	+			-	-	P,L,V	missense (SIFT:tolerated)	5:75659419
ARNTL2	*n*	-	-				P	upstream (3413 bp)	5:82942569
MGST1	*+*	+		-		-	P,V,D	upstream (4589 bp) intron	5:93954751 5:93945655
CSN2	*++++*		+				L,V,D	intron	6:87180731
CSN3	*++++*		-		-		P,L,D	missense (SIFT:tolerated) upstream (2036 bp)	6:87390576 6:87376362
GC	*n*	+	+		-	-	P,L,V	upstream (2582 bp)	6:88741762
RDH8	*n*	-					L	missense (SIFT:deleterious)	7:15815974
TTC7B	*+*			+			D	downstream (3086 bp)	10:103182221
PROM2	*++*	-					D	missense (SIFT:tolerated)	11:2003275
PAEP	*++++*	+	+			-	P,L,V,D	missense (SIFT:tolerated)	11:103303475
ABO	*++*		+				L,D	downstream (2688 bp)	11:104229609
DGAT1	*n*	+	+	-	-	-	P,L,V	intron missense (SIFT:tolerated)	14:1801116 14:1802266
COX6C	*n*		+	+			P,L	downstream (1091 bp) downstream (3684 bp)	14:66648812 14:66651404
TRIM29	*+++*	-				+	P, D	downstream (658 bp)	15:31212485
KRT19	*+++*	-		-			P,L,D	missense (SIFT:tolerated)	19:42366926
PTRF	*+*	-	-				P,D	upstream (4742 bp)	19:43166907
ERGIC1	*++*	-					L,D	intron	20:4543452
GHR	*+*	+					D,V	downstream (4947 bp)	20:31885789
SMEK1	*n*	+	+			-	P,V	downstream (2777 bp)	21:56798101
WARS	*+*	-	-				P,L,D	intron	21:66916247
MLH1	*n*	-					L,V	synonymous	22:10493668
GMDS	*+*	+					D	intron	23:51280200
MARF1	*n*	+	+				P	downstream (24 bp)	25:14138518
SCD	*+++*			+			D	downstream (1134 bp)	26:21140458
PRDX3	*n*		-	-			P,L	upstream (3744 bp)	26:39685136

The relative direction of the variant effect on milk traits is shown as ‘+’ or ‘-‘. The direction of effects for fat and protein percent (F%, P%) are included if their posterior probability was > 0.2 (AUS BayesRC Lact) as further validation of the Yield traits

^a^The strength of RNAseq differential gene expression in lactating mammary tissue compared to 17 other body tissues [22]. Differential expression is indicated if log2 fold change (LFC) > 1 (ie. >2^1^ increase in expression) and *p*-value < 1.0e-4 and “*n*” indicates no differential expression. The strength of expression is indicated as + for a LFC value between 1 to 2, ++ for 2 to 5, +++ for 5 to10 and ++++ for above 10

^b^Evidence for candidate genes included one or more of the following: a member of the Lact gene set (L), associated with more than one milk trait (P), differentially expressed in mammary tissue (D), and/or validated in the DANZ analysis (V)

**Table 9:** Full names of the candidate genes listed in Table 8

Official Gene Symbol	Gene Name
ROBO1	roundabout, axon guidance receptor, homolog 1 (Drosophila)
SLC37A1	similar to solute carrier family 37 member 1
PSMB2	proteasome (prosome, macropain) subunit, beta type, 2
OGDH	oxoglutarate (alpha-ketoglutarate) dehydrogenase (lipoamide)
MYH9	myosin, heavy chain 9, non-muscle
NCF4	neutrophil cytosolic factor 4
ARNTL2	aryl hydrocarbon receptor nuclear translocator-like 2
MGST1	microsomal glutathione S-transferase 1
CSN2	beta casein
CSN3	kappa casein
GC	group-specific component (vitamin D binding protein)
RDH8	retinol dehydrogenase 8 (all-trans)
TTC7B	tetratricopeptide repeat domain 7B
PROM2	prominin 2
PAEP	beta lactoglobulin
ABO	ABO blood group (transferase A, alpha 1-3-N-acetylgalactosaminyltransferase; transferase B, alpha 1-3-galactosyltransferase)
DGAT1	diacylglycerol O-acyltransferase homolog 1
COX6C	cytochrome c oxidase subunit VIc
TRIM29	tripartite motif containing 29
KRT19	keratin 19
PTRF	polymerase I and transcript release factor
ERGIC1	endoplasmic reticulum-golgi intermediate compartment 1
GHR	growth hormone receptor
SMEK1	SMEK homolog 1, suppressor of mek1
WARS	tryptophanyl-tRNA synthetase
MLH1	mutL homolog 1, colon cancer, nonpolyposis type 2
GMDS	GDP-mannose 4,6-dehydratase
MARF1	Meiosis arrest female 1(alias: KIAA0430)
SCD	stearoyl-CoA desaturase (delta-9-desaturase)
PRDX3	peroxiredoxin 3

## Discussion

This study demonstrates that the BayesRC method can simultaneously be used to map causal variants, to study genetic architecture and to predict future phenotypes as did Moser et al [2] and Kemper et al [3] for BayesR. However, our new BayesRC method is potentially more powerful than BayesR because it enables flexible integration of *a priori* biological information. We provided evidence that BayesRC can increase the accuracy of genomic prediction and QTL discovery compared to BayesR and GBLUP with informative prior biological information. We also showed that using imputed sequence data in coding regions increased prediction accuracy and power to detect rare causal variants compared to dense SNP array genotypes.

A desirable feature of Bayes RC is that the prior knowledge is incorporated objectively. In the case of GWAS for example, prior knowledge is only used post-analysis to confirm candidate genes. However, it is often possible to make a plausible case for many genes potentially affecting a trait. In Bayes RC, classes of sequence variants expected to differ in the proportion of variants having an effect on the trait are defined *a priori*. This leads to an objective estimate of the enrichment of effects within a class of variants. This enrichment is then used by the analysis in estimating the probability that any individual variant in the class has a non-zero effect.

BayesRC is somewhat similar to BayesRS [6] which uses prior knowledge of the variance explained by each segment of the genome, and then allocates a segment specific prior for the mixing proportions of variant effects expected in the four distributions. A key difference in BayesRC is that the prior is the same for the mixture proportions in all variant classes (i.e., a symmetric Dirichlet distribution). Thus the classes only differ in their estimated distribution of variant effects if this is supported by the data. Also, in BayesRC, the classification of variants to classes is flexible and straightforward to apply, incorporating information from a range of independent sources ranging from very broad to specific (such as lists of candidate genes, and known causal mutations). For example, in our BayesRC Lact analysis of simulated Trait 1, most variants in classes I and II were not QTL variants, but enrichment for QTL (13 % and 6 %) in these classes resulted in more power and precision than BayesR and GBLUP. Even when one third or one half of the 4000 QTL for Trait 1 were mis-assigned to class III (ie. reduced enrichment in Class I and II) the BayesRC model still showed the higher accuracy than BayesR (Additional file 1: Table S3). We used the Lact gene model to simulate 4000 QTL because previous studies have suggested that the number of loci affecting complex traits is at minimum several hundred up to several thousand [23–26]. Several studies have also provided strong evidence that within a QTL region, there are multiple alleles segregating that affect the trait (eg [23, 27]). However, provided that there is good prior biological information available, the BayesRC model should provide an advantage independent of the exact distribution of QTL effects.

In real dairy cattle data the increase in accuracy of genomic prediction with BayesRC was only modest at best. This is probably because the class I and II enrichment for non-zero effects was low (similar to the BayesRC Seq model for simulated Trait 2) and most of the genetic variance was explained by class III variants. For instance, for Milk Yield the proportion of variance explained by each class was approximately 6 % for class I, 13 % for class II and 81 % for class III. Thus to increase the accuracy of prediction, we need better prior biological information about the genes and sites in the genome that are likely to affect a particular trait. In human genetics the ENCODE annotations may well provide this [28]. Also, we expected the most difference between methods to be apparent in the Australian Red validation (least related to the training animals) but the lower reliability of these cow phenotypes may have partially masked real differences.

Another factor limiting prediction accuracy with Bayes RC in the real data is imperfect imputation of sequence and/or missing causal variants. In addition to imperfect imputation, we did not attempt to analyse full genome sequence but concentrated on gene coding regions, so undoubtedly a proportion of causal variants are missed. However, the use of imputed sequence variants in coding regions did generally increase the accuracy of genomic prediction in simulated and real data compared to high density SNP genotypes (Tables 4 and 5). In the real data it also enabled the discovery of a number of rare variants associated with milk traits that were not detected as QTL regions using only high density SNP genotypes: we gave two examples close to SMEK1 and CSH2 genes (Additional file 1: Figure S3 and S4).

SMEK1 to our knowledge has not been documented by other research groups as affecting milk production in dairy cattle and was not in our Lact gene set. However, it had a very high posterior probability and is a potential candidate gene because it is plays a regulatory role in the Insulin/IGF-1 signalling pathway [29] and is known to be involved in mammalian hepatic gluconeogenesis [30]. The Insulin/IGF-1 pathway influences key physiological processes related to mammary gland development such as cell proliferation and apoptosis. It is of course possible that the rare REG variant with the highest posterior probability (AUS analysis) may not be the actual causal mutation. A second REG variant, 1589 bp downstream from SMEK1, was excluded from our analysis because it was in perfect LD with our candidate REG variant. Also, both these REG variants are in very high LD (r^2^ > 0.75) with an NSC variant in SMEK1 which is predicted to have a deleterious effect on the protein (based on SIFT [31]).

CSH2 codes for a chorionic somatomammotropin hormone (a placental lactogen) which has been demonstrated to play a role in bovine mammogenesis and milk production [32] possibly by directly influencing the proliferation of luminal mammary cells [33]. Again, the variant identified in our study may not be the causal mutation, but could be in high LD with one regulating the expression of CSH2.

The candidate GC gene (Group-specific Component) in Fig. 5 encodes the vitamin D binding protein (VDBP) which is the main transporter of vitamin D in plasma. To our knowledge, no other independent studies have suggested this gene is associated with milk traits although it was included in our Lact gene set. The GC gene appears to be actively involved in the transport of vitamin D3: first transporting the sterol vitamin D3 from skin to liver, then its 25(OH)D3 derivative from liver to kidney and finally the active form, 1,25(OH)2D3, from kidney to the mammary gland and other tissues (reviewed by [34]). In vitro studies indicate that vitamin D3 is involved in regulating growth and differentiation of mammary epithelial cells [35–37] and these cells play a key role in determining the level of milk production.

The candidate gene MYH9 (Fig. 6) codes for a cellular myosin and to our knowledge has not been previously published as a candidate gene affecting milk traits, but was in our Lact gene list. It is known to play a role in the actin cytoskeleton and has been found to be highly expressed in terminal end buds of murine mammary tissue [38] implying a key role in mammary gland development. It may also be involved in controlling milk secretion through involvement in tight junctions [39]. Of the other two potential candidate genes for Milk Yield in Fig. 6, NCF4 was included in our Lact gene set while CSF2RB was not. However, CSF2RB was found to be highly over-expressed in lactating mammary tissue compared to 17 other tissues [40]. CSF2RB codes for the β subunit of cytokine receptors for the interleukin-3 family. The majority of cytokine receptors, in addition to playing a key role in immune signalling pathways, are involved in activation of the JAK/STAT pathway [41] which is known to be important for regulating mammary gland development.

The two NSC variants identified with the highest BayesRC Lact posterior probability in the PAEP (alias beta-lactoglobulin) gene (Fig. 7a) are the “causal mutations” that distinguish the well-known A and B forms of the beta-lactoglobulin protein in milk whey [20]. A number of studies have consistently found that animals homozygous for the A form of beta-lactoglobulin have higher concentrations of protein in their milk compared to those homozygous for the B form (eg. [20, 42–44]). Our results were in agreement with this: AA individuals having higher Protein Yields than the AB and BB individuals. Although the genetic basis of this effect has not yet been discovered, it is possible that there is a regulatory variant in strong LD with these two NSC variants (differentiating the A and B protein) which leads to increased transcription of the A form compared to the B form. The LD around the PAEP gene region is extremely high in our data, in keeping with the results of [20], possibly as a result of selection for protein yield in dairy cattle. We excluded 61 variants in a 10Kb region just upstream of PAEP from our analysis because they were in perfect LD with our highest probability variant. It is therefore possible that any one of these variants may be the causal mutation.

A number of the other candidate genes listed in Table 8 confirm previously documented examples of genes associated with milk traits including: CSN2 and CSN3 (both casein genes), DGAT1 and SCD (genes involved in fatty acid synthesis), MGST1, TTC7B (lipid metabolism) and GHR (growth hormone receptor) [45–52].

Some other genes in Table 8 that have not been previously documented as candidate genes for milk traits do fall in previously identified QTL regions. An example of this is KRT19 in which a NSC variant showed a strong association with Milk Yield and was also in our Lact gene set. This gene is one of a family of cytokeratins responsible for the structural integrity of epithelial cells and is one of a tight cluster of 3 keratin genes (KRT19, KRT15 and KRT17) all found to be highly over-expressed in lactating mammary tissue compared to 17 other tissues [40]. The KRT19 gene is a very plausible candidate gene because it potentially affects the integrity of mammary tissue, thereby indirectly affecting milk production. Some REG variants in Table 8 lie closest to the gene listed, but may in fact be associated with regulation of a different gene in the same region that affects the trait.

### Caveats

In theory it is possible to use many more classes in BayesRC than the three used here. However, although the biological priors are relatively uninformative, it is likely that the Dirichlet prior distribution may still have a moderately strong influence on the posteriors when the number of variants in one class is relatively low. When priors carry much uncertainty, such as our Lact classes, we recommend maintaining reasonable class sizes (more than 1000 variants) to ensure that the data has a strong influence on the posterior parameters. The main motivation for creating more than two classes should be the expectation that enrichment for QTL may differ between classes of variants.

A drawback of the BayesR and BayesRC methods is that they are computationally demanding, so it is important to develop faster Bayesian analytical approaches [53]. For 10,300 training individuals with ~994,000 SEQ variants and 40,000 MCMC iterations, a multi-threaded C++ version of the program took ~300 h per thread with ~80Gb memory (where each thread runs one of the replicate MCMC chains). Computation time increased approximately linearly with number of individuals and number of variants. Speed and Balding [54] proposed a very computationally efficient “MultiBLUP” method which differs from the standard GBLUP approach by allowing a mixture of normal distributions of SNP effects to be fitted similar to Bayesian approaches. The “biological priors” required for the MultiBLUP method are estimates of genome segment variance which are then used to partition variants into groups representing different expected effect-size variances. The authors reported an increased accuracy of genomic prediction compared to standard GBLUP particularly where some causal variants with a large effect were segregating. The prior biological information required for MultiBLUP is very similar to the requirements for BayesRS [6] but the former is likely to be considerably more computationally efficient. However, it is unlikely that MultiBLUP would show an advantage over standard GBLUP using our broad biological classifications because in one class there can be a wide range in the size of variant effects. Also, for QTL discovery, MultiBLUP would likely still show similar limitations as the standard GBLUP because the effect of a single true causal variant will tend to be spread over multiple SNP within segments.

If LD is very high across extended regions of the genome, and QTL effects are many and small, there is likely to be little difference between Bayesian and GBLUP genomic prediction when very dense markers are used [5]. We argue that for domestic species with small effective population sizes and resulting long-range LD, it is useful to combine data from more than one breed to reduce the strength of long-range LD. Also, prior filtering of sequence data helps to reduce the likelihood of finding extended regions of dense variants in strong LD with a single causal variant. The accuracy of genomic prediction using sequence variants will then persist better in less related individuals because QTL effect estimates are more precise (i.e., less likely to be spread across multiple variants in extended chromosome segments). Notably, our results demonstrate that when training and validation sets are very highly related there will be little difference in the observed accuracy between methods. Therefore, to expose the true precision of the QTL effect estimates, it is important to compare methods using validation sets which are not highly related to the training sets (Fig. 1).

## Conclusion

Our new BayesRC method provides a flexible approach to improving the accuracy of genomic prediction and QTL discovery, by taking advantage of prior biological knowledge that is already available for a range of traits and species. The approach used in BayesRC to incorporate biological priors is appealing because it is straightforward to apply and is incorporated objectively based on evidence from the data being analysed. Further research on discovering functional regions of the genome, as well as improving sequence and imputation accuracy of rare variant prediction are critical to realising the full potential of this and other similar methods.

### Data availability

The 1000 Bull Genomes Project (Run 2) has published the sequences of 129 Holstein and 15 Jersey bulls [40] that were used as our reference for sequence imputation. Project accession code (NCBI Sequence Read Archive - SRA), SRP039339 and run accessions; SRR1188706, SRR1262533, SRR1262536, SRR1262538, SRR1262539, SRR1262660 - SRR1262778, SRR1262780, SRR1262783, SRR1262785- SRR1262787, SRR1262789 – SRR1262803). An additional 19 sequences were included in our reference (12 Jersey and 7 Holstein) from Run3.0 1000 Bull Genomes Project. The list of 2.875 million sequence variants used for the analysis are available on request. The list of “Lactation” candidate genes are available in Additional file 2. BayesR code is available at: http://www.cnsgenomics.com/software/. The BayesRC compiled program is available on request for non-commercial research.

### Ethics statement

No experimental animal studies were conducted for the work detailed in this manuscript. References have been provided where animal data was used.

## Additional files

Additional file 1:
**Includes Supplementary Figures S1 to S4, Tables S1 to S4, and a summary of the independent experiments used to identify the "Lactation Gene Set".** (DOCX 1332 kb) Additional file 2:
**List of genes in the "Lactation Gene Set" that were used as our independent biological prior for the BayesRC Lact model.** (TXT 36 kb)
