# Supplementary material for: Exploiting biological priors and sequence variants enhances QTL discovery and genomic prediction of complex traits
Source: BMC Genomics. 2016 Feb 27;17:144. doi: 10.1186/s12864-016-2443-6 (PMC4769584; doi:10.1186/s12864-016-2443-6)
Supplement: Additional file 1: — Includes Supplementary Figures S1 to S4, Tables S1 to S4, and a summary of the independent experiments used to identify the "Lactation Gene Set". (DOCX 1332 kb) [file 12864_2016_2443_MOESM1_ESM.docx]

# Supplementary Materials

**Table S1**. **Sequence variants identified in coding and potentially regulatory regions from run 3.0 of the 1000 bull genome project pipeline**. Variants were grouped into three broad categories for our analysis: CHIP = high density SNP array variant, NSC=variants predicted to result in a non-synonymous coding change and REG=all variants in potentially regulatory regions (mainly within 5Kb of genes).

| **Annotation** | **Variant category** | **Number** | **Total** |
| --- | --- | --- | --- |
| intergenic_variant | CHIP | 368 265 |  |
| intron_variant | CHIP | 185 914 |  |
| synonymous_variant | CHIP | 4045 | **558 224** |
| coding_sequence_variant | NSC | 223 |  |
| frameshift_variant | NSC | 1366 |  |
| inframe_deletion | NSC | 954 |  |
| inframe_insertion | NSC | 318 |  |
| initiator_codon_variant | NSC | 210 |  |
| missense_variant | NSC | 113 282 |  |
| splice_acceptor_variant | NSC | 1554 |  |
| splice_donor_variant | NSC | 2323 |  |
| splice_region_variant | NSC | 1020 |  |
| stop_gained | NSC | 2910 |  |
| stop_lost | NSC | 98 |  |
| stop_retained_variant | NSC | 71 | **124 329** |
| 3_prime_UTR_variant | REG | 70 129 |  |
| 5_prime_UTR_variant | REG | 12 924 |  |
| downstream_gene_variant ^1^ | REG | 937 079 |  |
| mature_miRNA_variant | REG | 213 |  |
| nc_transcript_variant | REG | 86 |  |
| non_coding_exon_variant | REG | 10 299 |  |
| upstream_gene_variant ^1^ | REG | 1 072 157 | **2 102 887** |
| **TOTAL variants** |  |  | **2 785 440** |

^1^ Up- and down-stream variants were those within 5000 bp of a gene.

**Table S2.** **Linkage disequilibrium between 800K SNP or NSC variants with all other 800K SNP^a^.** The average number of SNP in high LD (r^2^ > 0.75) and the distance they span around each sampled variant is given for Holstein and Jersey breeds separately and in the combined breed data (Hol_Jer).

| Genotype pairs measured | ^a^ Statistic for LD r^2^ > 0.75 | Holstein | Jersey | Hol_Jer |
| --- | --- | --- | --- | --- |
| 800K SNP with 800K SNP | Average Number of Variants | 4.9 | 9.7 | 4.5 |
| 800K SNP with 800K SNP | Average Kb span | 67.8 | 280.4 | 67.2 |
| NSC variants with 800K SNP | Average Number of Variants | 1.8 | 4.1 | 1.7 |
| NSC variants with 800K SNP | Average Kb span | 69.6 | 217.1 | 47.5 |

^a^ Five hundred NSC and 500 800K variants were randomly sampled and LD (r^2^) was measured between these and all other 800K SNP. Using a threshold of r^2^ > 0.75, we present the average number and span (Kb) of 800K SNP variants in high LD with those in the randomly chosen samples. A random sample of 3900 cows of both Holstein and Jersey breeds was used to measure LD.

**Table S3**. **Accuracy and bias of genomic predictions for three simulated traits.** Four different validation sets were used that represented decreasing relatedness to the training population. Accuracy was estimated as the correlation between the genomic prediction and true genetic value, while bias was assessed by the regression coefficient of true genetic value on estimated genetic value.

|  |  | **Holstein Bull Only** | | **Holstein Bull & Cow** | | **Red Holstein** | | **Australian Red** | |
| --- | --- | --- | --- | --- | --- | --- | --- | --- | --- |
| Trait^b^ | Analysis  Model^a^ | Acc | Bias | Acc | Bias | Acc | Bias | Acc | Bias |
| 1 | BayesR 800K | 0.80 | 1.01 | 0.79 | 0.95 | 0.66 | 0.87 | 0.53 | 0.83 |
| 1 | BayesR SEQ | 0.86 | 1.06 | 0.84 | 0.98 | 0.73 | 0.91 | 0.63 | 0.88 |
| 1 | BayesRC Seq | 0.87 | 1.06 | 0.84 | 0.99 | 0.73 | 0.92 | 0.64 | 0.91 |
| 1 | BayesRC Lact | 0.90 | 1.05 | 0.90 | 1.00 | 0.81 | 0.96 | 0.79 | 1.03 |
| 1 | BayesRC 2/3Lact | 0.88 | 1.06 | 0.87 | 0.99 | 0.78 | 0.97 | 0.72 | 1.00 |
| 1 | BayesRC 1/2Lact | 0.88 | 1.07 | 0.86 | 1.00 | 0.75 | 0.93 | 0.69 | 0.97 |
| 1 | BayesRC RLact | 0.86 | 1.07 | 0.84 | 0.98 | 0.74 | 0.92 | 0.62 | 0.86 |
| 2 | BayesR 800K | 0.76 | 1.02 | 0.77 | 0.99 | 0.69 | 0.91 | 0.56 | 0.78 |
| 2 | BayesR SEQ | 0.84 | 1.06 | 0.83 | 1.00 | 0.76 | 0.98 | 0.69 | 0.93 |
| 2 | BayesRC Seq | 0.84 | 1.07 | 0.83 | 1.01 | 0.77 | 0.99 | 0.68 | 0.91 |
| 2 | BayesRC Lact | 0.84 | 1.06 | 0.83 | 1.00 | 0.76 | 0.98 | 0.68 | 0.92 |
| 2 | BayesRC 2/3Lact | 0.84 | 1.06 | 0.83 | 1.00 | 0.76 | 0.98 | 0.68 | 0.92 |
| 2 | BayesRC 1/2Lact | 0.84 | 1.06 | 0.83 | 1.00 | 0.76 | 0.97 | 0.68 | 0.92 |
| 2 | BayesRC RLact | 0.84 | 1.06 | 0.83 | 1.00 | 0.76 | 0.97 | 0.67 | 0.91 |
| 3 | BayesR 800K | 0.83 | 1.01 | 0.77 | 0.99 | 0.64 | 0.98 | 0.45 | 0.83 |
| 3 | BayesR SEQ | 0.87 | 1.01 | 0.82 | 1.01 | 0.66 | 0.97 | 0.60 | 1.07 |
| 3 | BayesRC Seq | 0.86 | 1.01 | 0.82 | 1.01 | 0.66 | 0.96 | 0.60 | 1.07 |
| 3 | BayesRC Lact | 0.86 | 1.00 | 0.82 | 1.01 | 0.66 | 0.96 | 0.60 | 1.07 |
| 3 | BayesRC 2/3Lact | 0.87 | 1.00 | 0.82 | 1.01 | 0.66 | 0.96 | 0.60 | 1.07 |
| 3 | BayesRC 1/2Lact | 0.86 | 1.01 | 0.82 | 1.01 | 0.66 | 0.97 | 0.60 | 1.07 |
| 3 | BayesRC RLact | 0.87 | 1.00 | 0.82 | 1.00 | 0.66 | 0.96 | 0.59 | 1.06 |

^b^  Three simulated traits were: 1) 4000 QTL simulated on variants in or close to a set of 790 Lact genes, 2) 4000 QTL simulated on NSC or REG variants only and 3) 4000 QTL simulated at random genome-wide. BayesR models were run with two genotype densities: 800K array and SEQ. Only the SEQ data contained the real QTL. The BayesRC models used SEQ data only and classes I, II and III were defined as below:

**BayesRC Lact** with variant classes:

I. NSC & in Lact genes

II. All variants other than NSC that overlap Lact gene regions (±50Kb)

III. All other SEQ variants not in class I or II

**BayesRC Seq** with variant classes:

I. NSC (non-synonymous coding)

II. REG (potentially regulatory)

III. CHIP (HD SNP chip variants not in the two previous classes)

**BayesRC 2/3Lact** – 1/3 of the Lact genes were replaced with randomly chosen genes

I. NSC & in 2/3Lact genes and 1/3 random genes

II. All variants other than NSC that overlap 2/3 of Lact gene regions and 1/3 random gene regions (±50Kb)

III. All other SEQ variants not in class I or II

**BayesRC 1/2Lact** – 1/2 of the Lact genes were replaced with randomly chosen genes

I. NSC & in 1/2Lact genes and 1/2 random genes

II. All variants other than NSC that overlap 1/2 of Lact gene regions and 1/2 random gene regions (±50Kb)

III. All other SEQ variants not in class I or II

**BayesRC RLact**

I. NSC & in a random set of 790 genes

II. Variants other than NSC that overlap a random set of 790 genes (±50Kb)

III. All other variants not in class I or II

## Genetic Architecture – AUS-Sim.

Table S4 compares the observed number of variant effects estimated for each of the four distributions in the BayesR and BayesRC analyses with the actual number of QTL simulated. The agreement was reasonable in most cases with SEQ genotypes (QTL in the data).

In Trait 1 (causal variants simulated on variants in/near Lact genes) the BayesRC Lact model gave the best estimate of the number of QTL per distribution, while there was considerable underestimation in other models. One potential reason for the bias in these other models is that many of the QTL were simulated on NSC and REG variants. This reduces the power to detect the QTL because these variants are often at lower allele frequency than 800K variants (and therefore in low LD with 800K SNP) and they segregate less frequently in both breeds than 800K variants. The power to detect individual causal variants may also have been compromised because QTL were restricted to 790 gene coding regions so in some cases two individual QTL may be in strong LD and may be hard to distinguish from a single QTL effect. In the BayesRC Lact model more of the true QTL effects had a higher probability of being detected because they were allocated to QTL enriched classes I and II.

For Trait 2, where causal mutations were only simulated on NSC and REG variants, there was some underestimation of the total number of QTL for all SEQ models. The main reason for this is likely to be that the causal variants were limited to the less common NSC or REG variants, reducing the power to detect these rarer QTL. For trait 2 the estimated number of QTL closest to the true value was found in the BayesRC Seq analysis where QTL were correctly assigned to NSC and REG classes, even though this represented relatively weak enrichment of QTL in these classes (3% and 0.5% respectively).

For Trait 3, QTL were simulated randomly genome-wide, many on 800K SNP, and there was some overestimation of the total number of QTL. The bias was specific to the smallest variance distribution (0.0001𝜎^2^_g_) and was apparent even in the SEQ models where all causal variants are in the data. This is likely due to some causal variants being in very strong LD with several non-causal variants, resulting in the latter being included in the model and most often being allocated to the smallest variance distribution (0.0001𝜎^2^_g_).

**Table S4**. **Average number of variant effects estimated in each of the four variance distributions for a range of BayesR and BayesRC analyses.** The true number of QTL simulated per distribution is shown in the top row of the table (bold font).

|  |  | **Average Number of Variants in each Distribution** | | | |  |
| --- | --- | --- | --- | --- | --- | --- |
| **Trait^a^** | **Model** | **0.0𝜎^2^_g_** | **0.0001𝜎^2^_g_** | **0.001𝜎^2^_g_** | **0.01𝜎^2^_g_** | **Total number of non-zero SNP effects** |
| **All traits** | **True Number of simulated QTL** | - | **3485** | **500** | **15** | **4000** |
| 1 | **BayesR 800K** | 382679 | 1521 | 494 | 19 | 2034 |
| 1 | **BayesR SEQ** | 918353 | 1629 | 533 | 19 | 2182 |
| 1 | **BayesRC Seq** | 917863 | 2161 | 490 | 20 | 2671 |
| 1 | **BayesRC Lact** | 916945 | 3175 | 393 | 21 | 3589 |
| 1 | **BayesRC RLact** | 918318 | 1666 | 528 | 22 | 2216 |
| 2 | **BayesR 800K** | 381886 | 2418 | 386 | 23 | 2827 |
| 2 | **BayesR SEQ** | 917202 | 2958 | 346 | 28 | 3332 |
| 2 | **BayesRC Seq** | 916781 | 3408 | 313 | 32 | 3753 |
| 2 | **BayesRC Lact** | 917231 | 2912 | 362 | 29 | 3303 |
| 2 | **BayesRC RLact** | 917207 | 2943 | 355 | 30 | 3327 |
| 3 | **BayesR 800K** | 380637 | 3593 | 475 | 9 | 4076 |
| 3 | **BayesR SEQ** | 916125 | 3944 | 447 | 18 | 4409 |
| 3 | **BayesRC Seq** | 916285 | 3772 | 454 | 23 | 4249 |
| 3 | **BayesRC Lact** | 916061 | 4010 | 441 | 21 | 4473 |
| 3 | **BayesRC RLact** | 916204 | 3850 | 460 | 20 | 4330 |

^a^  Three simulated traits were:

1. QTL simulated on variants in or close to a set of 790 Lact genes,
2. QTL simulated on NSC or REG variants only
3. QTL simulated at random genome-wide. See Table S3 for a description of BayesRC models.

## Lactation Gene Set

The “Lact” gene set was created from the results of eight independent heterogeneous microarray experiments performed in a collaboration between the former Department of Primary Industries, Victoria, Australia and AgResearch, New Zealand [full details in 1]. These were cDNA microarray experiments that examined gene expression changes in the bovine mammary gland during lactation, under two or more sets of experimental conditions that affected levels of milk production. A key objective of this microarray analysis was therefore to determine if there were gene expression patterns associated with changes in milk production. The eight independent studies are very briefly outlined below:

1) In vivo bromocriptine-mediated prolactin loss experiment. Comparing pregnant cows treated with bromocriptine prior to calving to those that were not.

2) In vitro bovine mammary epithelial cells in response to prolactin and extracellular matrix. Primary mammary epithelial cells harvested from 3 pregnant Holstein/Friesian heifers were cultured on either tissue culture plastic (TCP) or on Matrigel^®^, a commercial preparation of extracellular matrix (ECM) (BD Biosciences, USA), with or without the addition of prolactin.

3) Extended lactation program in cows examining the difference between:

a. Early and late lactation

b. Persistent and non-persistent dairy cows

c. Cows in extended lactation with low, medium or high nutritional status

4) Genetic merit experiment: comparing cows of high and low genetic merit under normal conditions, when fasted for 36 hours and when on 50% rations for 7 days.

5) Time course involution experiment involving the induction of involution by termination of milking and transcript profiling at 6, 12, 18, 24 and 36 hours post-milking. Full methods for this experiment are described in [2].

6) Streptococcus uberis-induced mastitis experiment. Full methods for this experiment are described in [3].

7) Once a day milking versus twice daily milking experiment.

8) Growth hormone treatment experiment. Hayashi et al describe the animals and treatment in detail [4].

Independent experimental validation of the microarray expression results was performed by Northern analysis and quantitative real-time PCR for a subset of significantly differentially regulated expressed sequence tags (ESTs) and for ESTs representing housekeeping genes. Genes corresponding to ESTs with a minimum fold change value of 1.5 and P<0.1 in two or more of these microarray experiments were identified and added to the “Lact” gene set.

The list of Lact genes are provided in Additional file 2.

## References

1. Vander Jagt CJ. Identifying genes critical to milk production. 2012; PhD Thesis. University of Melbourne.
2. Singh K, Davis SR, Dobson JM, Molenaar AJ, Wheeler TT, Prosser C et al. cDNA Microarray Analysis Reveals that Antioxidant and Immune Genes Are Upregulated During Involution of the Bovine Mammary Gland. J Dairy Sci. 2008; 91:2236-46.
3. Swanson KM, Stelwagen K, Dobson J, Henderson HV, Davis SR, Farr V et al. Transcriptome profiling of Streptococcus uberis-induced mastitis reveals fundamental differences between immune gene expression in the mammary gland and in a primary cell culture model. J Dairy Sci. 2009; 92:117-29.
4. Hayashi A, Nones K, Roy N, McNabbW, Mackenzie D, Pacheco D et al. Initiation and elongation steps of mRNA translation are involved in the increase in milk protein yield caused by growth hormone administration during lactation. J Dairy Sci. 2009; 92:1889-99.

**D.**

**E.**

**F.**

**C.**

**B.**


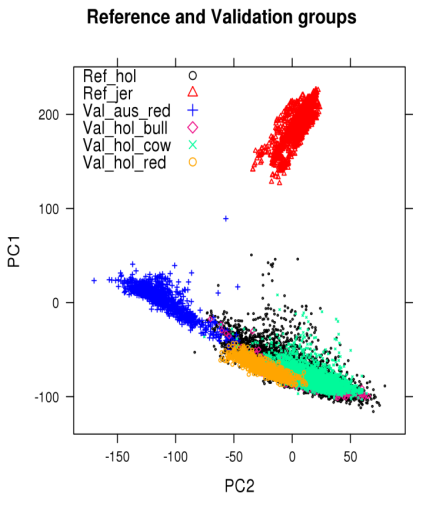

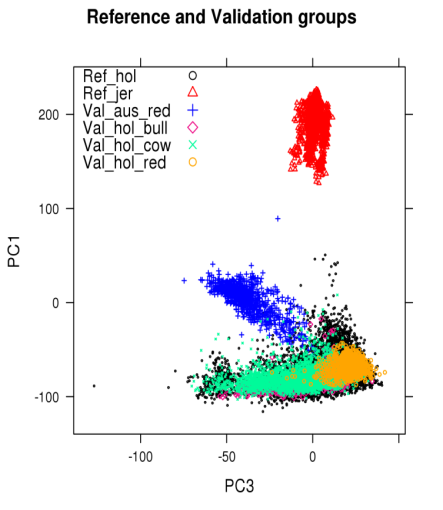

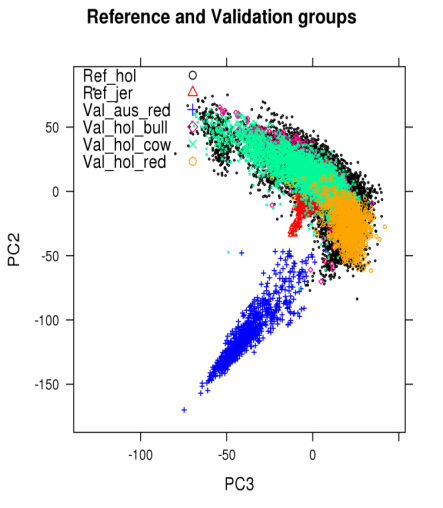

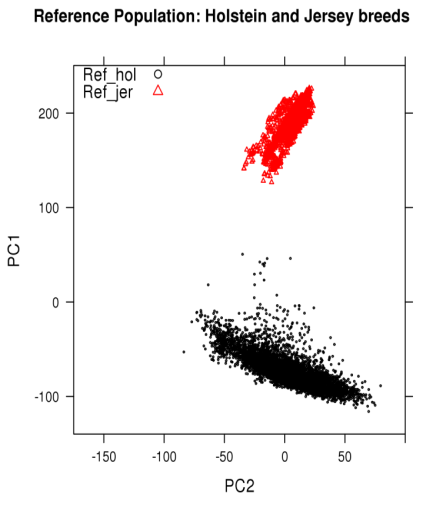

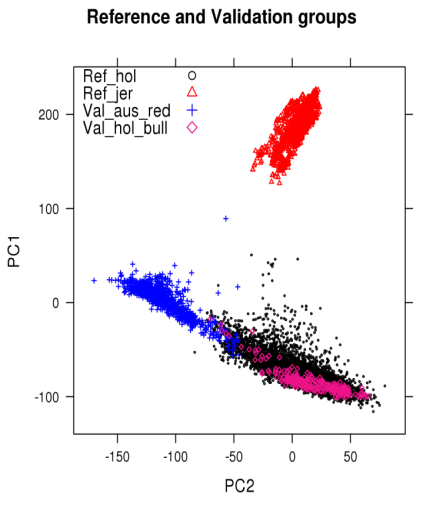

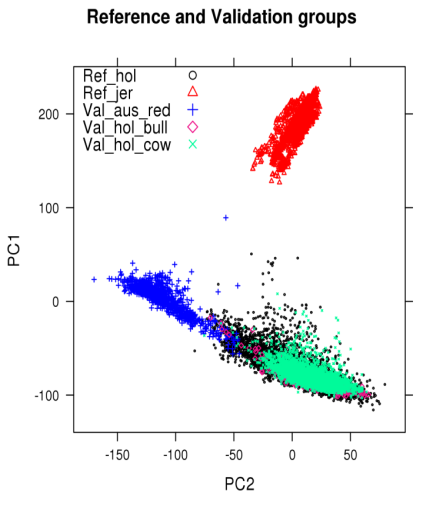


**A.**

**Figure S1.** **Principal components (PC) analysis of genomic relationships (using R software)**. The analysis was based on a genomic relationship matrix constructed from 50K array SNP genotypes. PC1 and PC2 are used to visualise genetic grouping among reference (training) animals (A) and the different validation groups (B, C and D). E shows all reference and validation groups (as in D) for PC1 and PC3, while F shows PC2 and PC3.


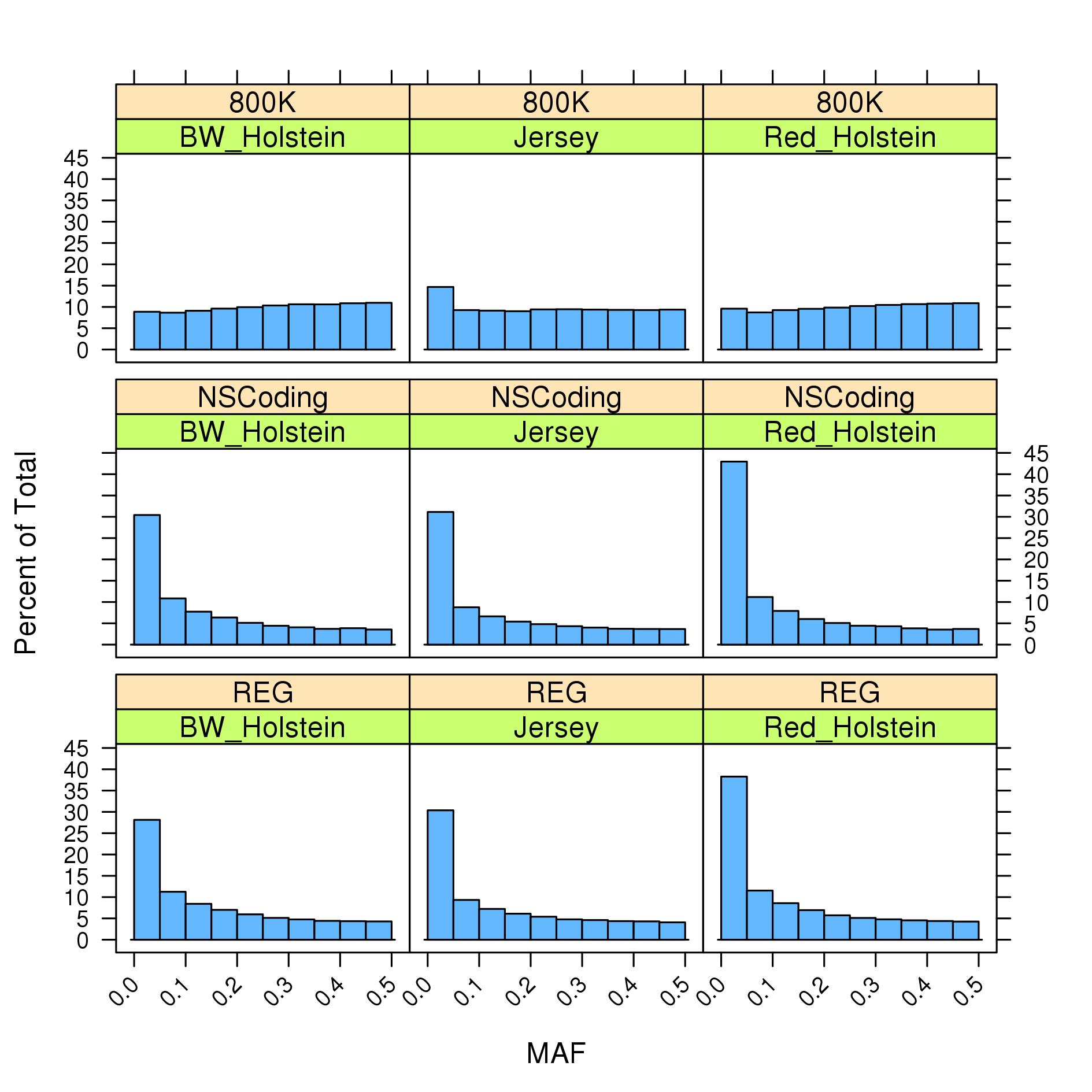


**Figure S2. Spectra of minor allele frequencies (MAF).** Each variant group are shown separately (non-synonymous coding =NSCoding, potentially regulatory (within 5Kb of genes)=REG and 800K= HD SNP array genotypes) within black and white Holstein (BW_Holstein), Red Holstein and Jersey breeds.


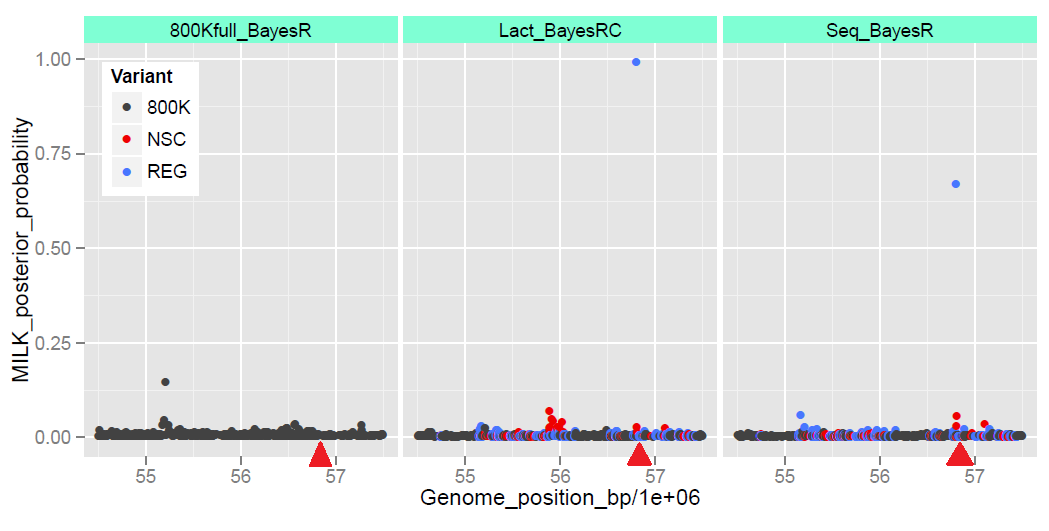
**Figure S3.** **Discovery of a SEQ REG variant showing a very high posterior probability for Milk Yield in the BayesRC Lact and BayesR SEQ analyses.** In the BayesR 800K analysis there was no apparent association with milk Yield in this region. The variant lies very close to the SMEK1 gene marked with the red triangle.


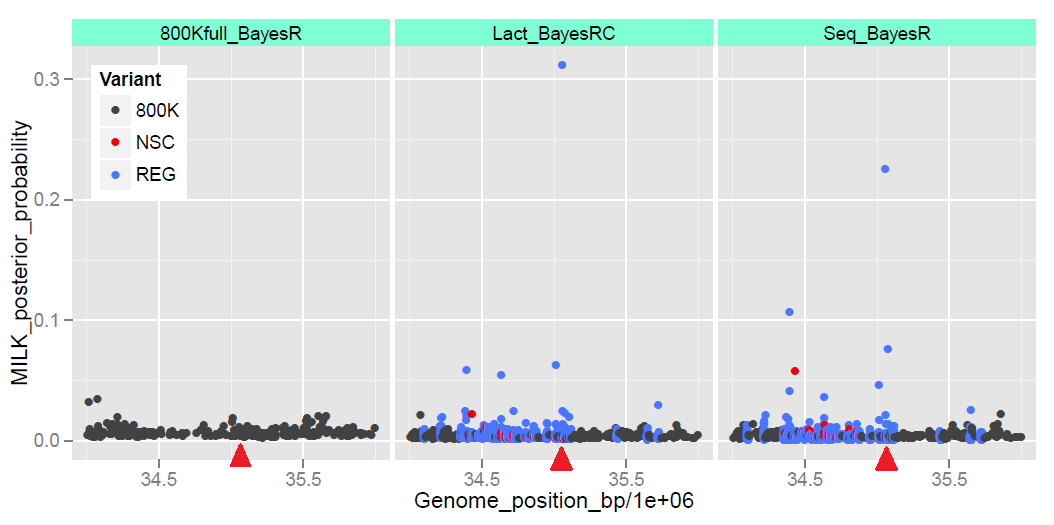


**Figure S4.** **Discovery of a SEQ REG variant showing a high posterior probability for Milk Yield in the BayesRC Lact and BayesR SEQ analyses.** In the BayesR 800K analysis there was no apparent association with milk Yield in this region. The variant lies very close to the CSH2 gene marked with the red triangle.
